# Risk period for transmission of SARS-CoV-2 and seasonal influenza: a rapid review

**DOI:** 10.1017/ice.2025.11

**Published:** 2025-03

**Authors:** Erin C. Stone, Devon L. Okasako-Schmucker, Joanna Taliano, Melissa Schaefer, David T. Kuhar

**Affiliations:** 1 Hubert Department of Global Health, Laney Graduate School, Emory University, Atlanta, GA, USA; 2 Prevention and Response Branch, Division of Healthcare Quality Promotion, National Center for Emerging and Zoonotic Infectious Diseases, Centers for Disease Control and Prevention, Atlanta, GA, USA; 3 Office of Science Quality and Library Services (OSQLS), Office of Science (OS), Centers for Disease Control and Prevention (CDC), Atlanta, GA, USA

**Keywords:** Viral, Respiratory illness, Omicron, Influenza, Rapid review, Symptomatic infectious period, Serial interval, Shedding, Symptoms

## Abstract

**Background:**

Restricting infectious healthcare workers (HCWs) from the workplace is an important infection prevention strategy. The duration of viral shedding or symptoms are often used as proxies for the infectious period in adults but may not accurately estimate it.

**Objective:**

To determine the risk period for transmission among previously healthy adults infected with SARS-CoV-2 omicron variant (omicron) or influenza A (influenza) by examining the duration of shedding and symptoms, and day of symptom onset in secondary cases of transmission pairs.

**Design:**

Rapid review

**Methods:**

This rapid review adhered to PRISMA-ScR; five databases were searched. The cumulative daily proportion of participants with an outcome of interest was calculated for each study and summarized.

**Results:**

Forty-three studies were included. Shedding resolved among ≥ 70% of participants by the end of day nine post symptom onset for omicron, and day seven for influenza; and for ≥ 90% of participants, by the end of day 10 for omicron and day nine for influenza. Two studies suggested shedding continues > 24 hours post-fever resolution for both viruses. Symptom onset occurred in ≥ 80% of secondary cases by the end of day seven post-primary case symptom onset for omicron and day six for influenza.

**Conclusions:**

Omicron shedding is consistent with previous recommendations to exclude infected HCWs from work for 10 days; and influenza follows a similar trend. Earlier symptom onset in most secondary cases for both pathogens indicates that, despite persistent viral shedding, most transmission occurs earlier; and the cumulative serial interval might better approximate the duration of infectiousness.

## Introduction

Healthcare workers (HCWs) can serve as a source of transmission of severe acute respiratory syndrome coronavirus 2 (SARS-CoV-2) and seasonal influenza in healthcare settings. Restricting infected HCWs from work while contagious is a mainstay of preventing transmission and maintaining a safe work and patient care environment.^1–3^ However, restricting HCWs from work has the potential to cause harm such as staffing shortages that can result in lapses in HCW safety and suboptimal patient care.^4–7^ Therefore, it is important to exclude HCWs from work for long enough to mitigate risk for respiratory virus transmission while minimizing any unintended health and safety consequences for HCWs and patients.

Criteria for returning infected HCWs to work include the duration of infectious period, availability of other workplace controls (e.g., masking) that might diminish risk for transmission, and consequences of excluding HCWs from work. The daily risk for transmission from a contagious HCW approximates resolution of the infectious period and informs the duration of exclusion from work. However, this is difficult to define precisely because measures typically used to approximate the infectious period may not accurately convey the duration of risk for transmission to others. For example, the duration of viral shedding can be used to approximate how long an individual may be contagious; however, some tests like the nucleic acid amplification test might detect noninfectious virus resulting in overestimates of the contagious period and duration of work restrictions.^8^

**Figure 1.**
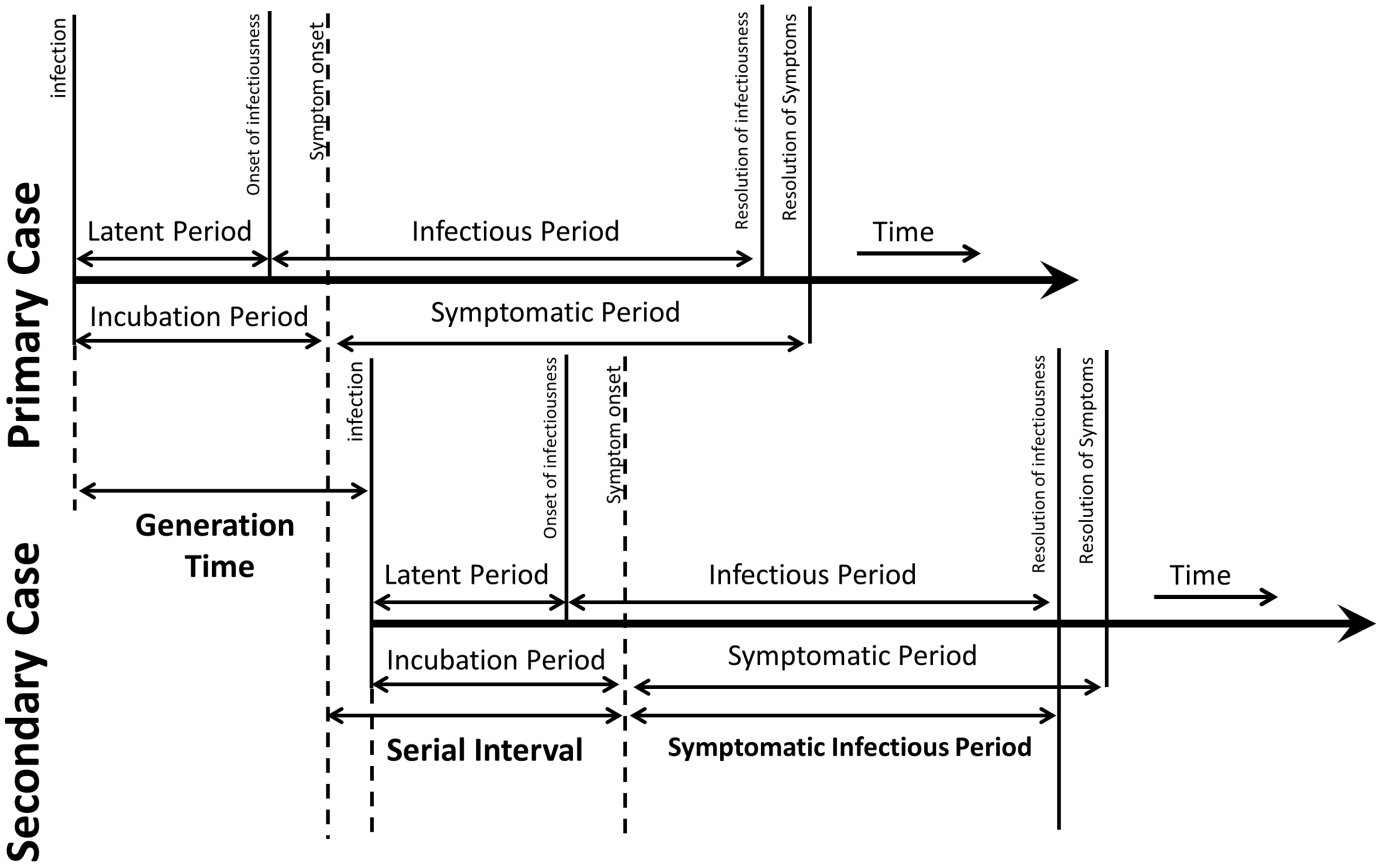
Transmission parameters (adapted from Kim et.al. 2023)^9^.

Transmission studies often approximate the time to infection in a secondary case using generation time, the time from the moment of infection in a primary case to infection in a secondary case (Figure 1, adapted^9^), which is impractical to accurately measure in a study because the moment of infection is often unknown. Serial interval, the time from symptom onset in a primary case to symptom onset in a secondary case, can be measured in a study, and is often substituted for generation time. Although a person’s contagious period may begin during the incubation period (the time between exposure and symptom onset), most only realize they are contagious when symptoms begin and, in these cases, it is important to know the duration of the contagious period after symptom onset. Hence, subtracting the incubation period from the serial interval could approximate the symptomatic contagious period—the period from symptom onset to end of contagiousness – and inform work restrictions. The objective of this rapid review was to estimate the daily risk for transmission from symptomatic HCWs by assessing the literature on the duration of viral shedding and serial intervals for SARS-CoV-2 omicron variant (omicron) or influenza A (influenza).

## Methods

To retrieve the influenza and SARS-CoV-2 publications most relevant to HCWs, this review focused on the most recent SARS-CoV-2 variant, omicron, and the most commonly occurring type of seasonal influenza, influenza A. This rapid review is reported according to the Preferred Reporting Items for Systematic reviews and Meta-Analyses extension for Scoping Reviews (PRISMA-ScR) guidelines.^10^

### Search strategy & study selection

The research questions guiding this review were: Among previously healthy symptomatic adults infected with mild SARS-CoV-2 (omicron variant) or influenza A:

1. What is the duration of viral shedding measured from symptom onset or diagnosis using culture or quantitative reverse transcription polymerase chain reaction (RT-qPCR)?
2. What is the association between the resolution of symptoms, specifically fever, and the resolution of viral shedding measured using culture, RT-qPCR, or reverse transcription polymerase chain reaction (RT-PCR)?
3. What is the pair-level serial interval, defined as the number of days between symptom onset in primary and secondary cases?

An information specialist (J.T.) developed research question-specific search strategies for omicron and adapted each strategy for influenza. The searches were performed in MEDLINE, Embase, Cochrane Library, CINAHL, and Scopus from the start of each database to July 26 or August 1, 2024 (Tables s4-s9). Results of the literature searches were uploaded into EndNote 21 (Clarivate Analytics©, Thomson Reuters, New York, NY, USA), where duplicates were removed, and two reviewers screened all titles and abstracts and subsequent full texts using exclusion criteria selected to reduce pre-identified risks of bias in this literature base. (Full selection criteria are in Supplementary Material Table s1.)

### Extraction

Data were extracted by two reviewers using a standardized Microsoft Excel (2021) form, and differences were reconciled by discussion. Extracted data included study and population characteristics, and the outcomes of interest (Table s2). For research question 3, serial intervals of zero or less were not extracted. When interventional studies met inclusion criteria, only placebo group data were extracted. Outcome data were extracted as presented in the studies or calculated using existing values. When data were not provided, it was abstracted from relevant figures using an online digitizing application (PlotDigitizer Online App).^11^ Potential areas for risk of bias were identified *a priori* to guide study selection, and possible confounding factors were identified for use in data analysis; thus, risk of bias assessments of studies were not conducted.

### Analysis

Shedding and symptom outcomes were analyzed from symptom onset, diagnosis, or inoculation (for influenza challenge studies) in the participant. Serial interval outcomes were analyzed from symptom onset in the primary case, which was defined as day zero. The cumulative proportion of participants with the outcomes of interest were calculated for each study using Microsoft Excel (2021) and R Studio. Analysis for risk stratification was conducted for study-level cumulative proportions of 70% – 100% of participants experiencing resolution of shedding for individuals or symptom onset in secondary cases. The days examined included day four through the current CDC recommended duration of work restrictions for HCWs with SARS-CoV-2 infection (Day 10 post symptom onset).^2^ Stratified analyses were performed to visually inspect the influence of confounding factors. These factors included household transmissions compared with community transmission; collectivist compared to individualist societies to approximate mask usage;^12^ vaccination status; viral sub-variant or subtype; and non-pharmaceutical interventions (NPI) such as masking and lockdown policies. Complete methods are found in the Supplementary Material.

## Results

**Figure 2.**
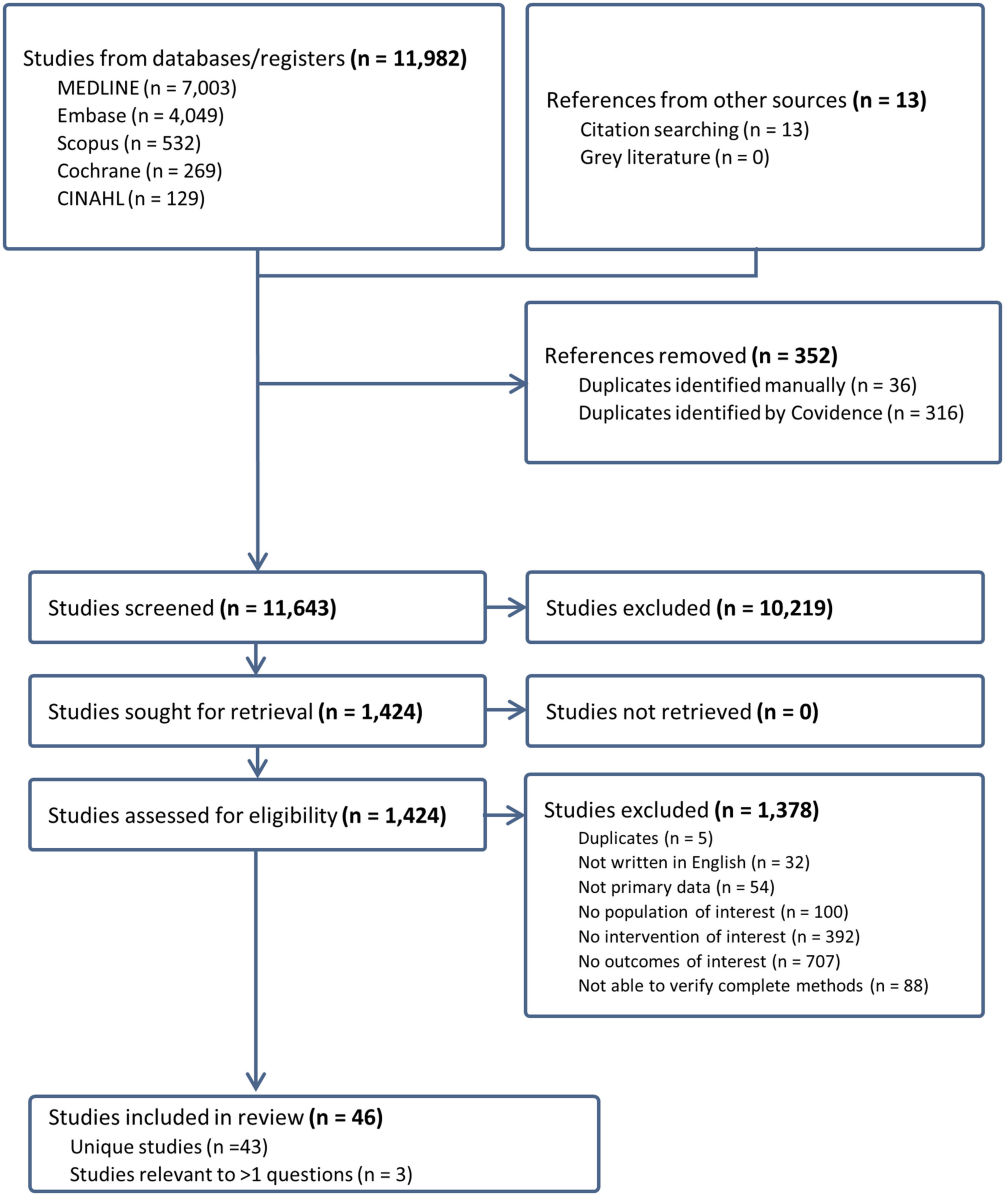
Flow chart of all studies.

Six search strategies identified a total of 11,995 titles and abstracts. After removal of 352 duplicates, 11,643 titles and abstracts were screened and 10,219 were determined to be not relevant to any research question. 1,424 full-text articles were assessed, of which 43 unique studies met inclusion criteria. Three studies were relevant to more than one research question: RQ1 (shedding) and RQ2 (shedding and symptoms). The total number of participants was 16,855 (range: 8 - 11,512). Over half of these studies (52%) were conducted in the United States, China, and South Korea, and 28% in the global south. The flowchart of combined search results contains duplicate articles that were screened uniquely in each of the six searches (Figure 2). All PRISMA diagrams (Figure s7–s12) and study characteristics (Table s3) are found in the Supplementary Material.

### Shedding

**Figure 3.**
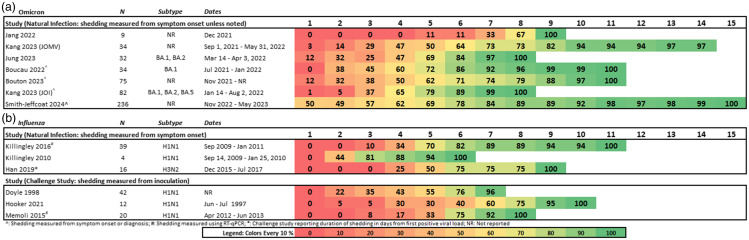
The cumulative proportion (%) of participants whose shedding resolved, measured in days from symptom onset, diagnosis, or inoculation. A. Studies reporting omicron. B. Studies reporting influenza.

Thirteen studies reported the duration of viral shedding for omicron or influenza measured daily using RT-qPCR (for influenza)^13^ or culture^8,14–24^ measured from symptom onset^19–21^ or both symptom onset and diagnosis.^8,14,15,22^ Seven studies reported duration of shedding for omicron,^8,14,15,19–22^ and three of these studies reported the sub-variant including BA.1,^14,20,21^ BA.2,^20,21^ and BA.5.^21^ Six studies reported duration of shedding for influenza:^13,16–18,23,24^ three reported shedding measured from symptom onset during natural infections^17,23,24^ with the H1N1 subtype,^23,24^ or the H3N2 subtype;^17^ and three challenge studies^13,16,18^ reported shedding measured from inoculation with H1N1. Figure 3 provides the daily cumulative proportion of resolution of omicron or influenza shedding.

By the end of day four, no omicron studies reported resolution of shedding among at least 70% of participants, but by the end of day nine, all studies (N = 7, 502 participants) reported resolution of shedding among ≥ 70% of participants with omicron (Table 1). No studies exceeded the ≥ 90% threshold by the end of day six; however, all omicron studies reported resolution of shedding among ≥ 90% of participants by the end of day 10. The influenza data suggests a shorter time to resolution of shedding. By the end of day four, one small influenza study^17^ (N = 16) reported resolution of shedding among ≥ 70% of participants, and by the end of day nine, all six influenza studies (N = 133) reported resolution of shedding among ≥ 90% of participants. Three omicron studies reported daily shedding data among either unvaccinated, vaccinated, or vaccinated and boosted participants^14,15,25^ (Figure s1). Vaccinated participants received either a single vector or two messenger RNA (mRNA) vaccines, and boosted participants received the full vaccination plus a subsequent dose. Two studies^14,25^ suggested unvaccinated participants shed longer than vaccinated participants, however the number of days is unclear (range 1–3 days). Two studies^14,15^ stratified duration of shedding for vaccinated and boosted participants, results were inconsistent and inconclusive. No influenza studies meeting inclusion criteria reported comparisons of shedding data among vaccinated and unvaccinated participants. There was not a clear difference in duration of shedding when comparing omicron studies that measured from symptom onset compared with symptom onset and diagnosis, or when comparing influenza studies that measured using RT-qPCR^13,17^ or culture. The three influenza challenge studies^13,16,18^ reported a lower proportion of participants with resolved shedding on day five, when compared with the three natural infection studies;^17,23,24^ and all three challenge studies reported resolution of shedding among 100% of participants one day earlier than the three natural infection studies.

**Table 1.** Daily Cumulative number of studies reaching at least a cumulative threshold of (A) participants with resolution of shedding measured in days from symptom onset, diagnosis, or inoculation, or (B) secondary cases with symptom onset in secondary cases measured in days from primary case symptom onset

Virus, N Studies (N participants/pairs)	Cumulative Participant Threshold (%)	Cumulative number of studies (Participants in study)						
		Day 4	Day 5	Day 6	Day 7	Day 8	Day 9	Day 10
A. Participants with resolution of shedding								
Omicron 7 (502)	70	0	2 (157)	5 (459)	6 (493)	6 (493)	7 (502)	
	80	0	0	3 (189)	4 (425)	4 (425)	7 (502)	
	90	0	0	0	3 (189)	3 (189)	4 (198)	7 (502)
	100	0	0	0	0	2 (114)	3 (123)	3 (123)
Influenza 6 (133)	70	1 (16)	2 (55)	5 (113)	5 (113)	6 (133)		
	80	1 (16)	1 (16)	2 (55)	4 (109)	4 (109)	6 (133)	
	90	0	1 (16)	1 (16)	3 (70)	3 (70)	6 (133)	
	100	0	0	1 (16)	1 (16)	3 (70)	3 (32)	5 (94)
B. Secondary cases with symptom onset								
Omicron, 14 (≥ 14,873)^a^	≥ 70	12 (≥ 14,317)	14 (≥ 14,873)					
	≥ 80	8 (≥ 772)	12 (≥ 14,317)	13 (≥ 14,430)	14 (≥ 14,873)			
	≥ 90	1 (48)	5 (377)	11 (≥ 2,584)	12 (≥ 14,332)	14 (≥ 14,873)		
	≥ 100	0	0	2 (214)	4 (235)	5 (510)	7 (638)	10 (≥ 969)
Influenza, 14 (303)	70	14 (303)						
	80	10 (166)	13 (252)	14 (303)				
	90	4 (52)	11 (175)	13 (252)	14 (303)			
	100	3 (33)	5 (46)	9 (155)	11 (180)	12 (194)	13 (252)	13 (263)

^a^1 study (an der Heiden, 2022) reported the denominator as household clusters, leaving the total number of secondary cases unclear^35^.

### Shedding and symptoms

Four included studies reported a daily assessment of symptoms and shedding measured via culture,^17,19^ RT-qPCR,^13^ and RT-PCR.^26^ The duration of shedding exceeded the duration of fever in the two studies reporting daily measurements among participants infected with omicron^19^ or influenza (Figure s2).^26^ Specifically, one small study^19^ (N = 8) reported five of eight omicron patients with fever experienced culture-positive shedding > 24 hours (range 1-4 days) after fever resolution and resolution of shedding occurred by the end of day 6-8 post-symptom onset in these participants. One influenza study^26^ found that 55% of participants with the H1N1 subtype were still shedding three days beyond fever resolution, and 17% were shedding by the end of day six post-resolution of fever when measured using RT-PCR. For participants with seasonal influenza, the duration of shedding following fever resolution was shorter, with 70% of participants shedding by the end of day three and only 3% shedding by the end of day five post-fever resolution (Figure s2). One influenza challenge study^13^ reported that among 20 participants with H1N1, two (10%) experienced resolution of shedding occurring one- and two-days post-symptom resolution; and another H1N1 challenge study^17^ did not report individual data and shedding beyond resolution of symptoms could not be ascertained. However, in aggregate, shedding measured via culture peaked by the end of day one post-inoculation and ended for all participants by the end of day six post inoculation, while symptoms peaked by the end of day three post-inoculation and ended on day 11 for some participants.

### Secondary case symptom onset

**Figure 4.**
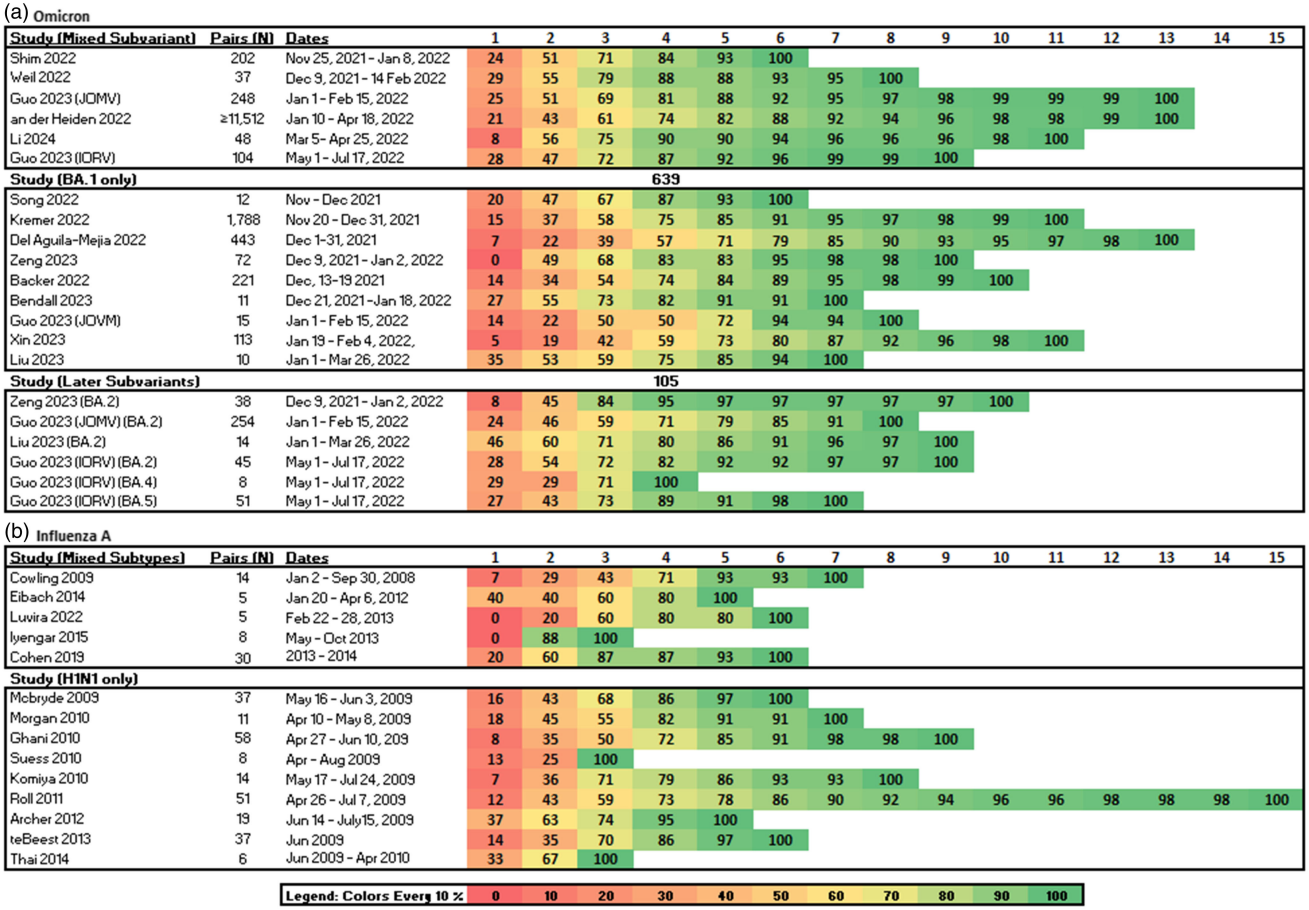
The cumulative proportion (%) of symptom onset in secondary cases measured in days from symptom onset in the primary case. A. Studies reporting omicron. B. Studies reporting influenza.

Twenty-eight studies reported the serial interval for transmission pairs, households, or cases. Fourteen reported serial interval data for omicron^27–40^ (Figure 4A), and fourteen for influenza^41–54^ (Figure 4B). All but three studies across both viruses reported that at least 50% of secondary cases experienced symptom onset by the end of day three following symptom onset in the primary case (Figure 4).

In all studies reporting any sub-variant of omicron, symptom onset occurred in at least 70% of secondary cases by the end of day five post symptom onset in the primary case (Table 1). Among studies reporting influenza transmission, secondary case symptom onset occurred in at least 70% of secondary cases in all studies by the end of day five, and by the end of day six for H1N1 (Figure 4).

Symptom onset occurred in at least 80% of omicron secondary cases by the end of day five for all studies reporting mixed sub-variants, day six for later sub-variants, and day seven for studies reporting BA.1. Secondary case symptom onset occurred in at least 80% of transmissions by the end of day five for all studies reporting mixed influenza subtypes, and by the end of day six for H1N1 (Figure 4).

In studies reporting transmission of mixed omicron sub-variants or BA.1, secondary case symptom onset did not reach 100% until the end of day 10; however, 100% of secondary case symptom onset occurred by the end of day nine for later sub-variants (BA.2, BA.4, and BA.5). While symptom onset occurred for all secondary influenza cases by the end of day seven for all mixed subtype studies, this milestone was not reached until day 15 for H1N1 infections (Figure 4). Studies reporting omicron variants were stratified by vaccination status of the population, the country- or region-level implementation of societal NPI such as lockdown and universal masking, masking as a cultural norm, and household vs. non-household transmissions; there was no difference in results (Figure s3–s6). There were insufficient data to stratify influenza studies by the implementation of NPIs.

## Discussion

The comparison of resolution of shedding with resolution of symptoms, and the serial interval, for influenza A and omicron infections help to better understand how the risk for transmission changes over time. The shedding data for omicron are consistent with previous return-to-work recommendations that exclude infected HCWs from work for up to 10 days; and the viral shedding for influenza followed a similar trend and resolved approximately a day sooner than for omicron. For both influenza and omicron, viral shedding persisted after fever resolved and other symptoms began to improve. However, earlier symptom onset in the vast majority of secondary cases for both pathogens suggests that, despite persistent viral shedding, most transmission occurs earlier.

Symptom onset in a secondary case likely does not indicate the day of transmission. Although transmission could occur before the onset of symptoms in the primary case, HCWs will generally not be recognized as potentially infectious before symptom onset unless asymptomatic testing is being performed. In routine practice, the period for which a HCW is restricted from work typically begins on the date of their symptom onset—or on the date of a first positive test – and the end date could be based on the duration of viral shedding or potentially when an acceptable level of residual risk for transmission has been reached, and this interval needs to be approximated (Figure 1). If using the date of secondary case symptom onset as a proxy for the date of transmission, it is important to recognize that actual transmission took place before the date of symptom onset. The incubation period represents time between the moment of transmission and symptom onset and can be used to estimate the time of transmission to a secondary case. Subtracting the shortest possible incubation period for influenza or omicron from the day of symptom onset in a secondary case might estimate the latest likely day that transmission occurred. The incubation period for influenza and omicron have been estimated to be 1.3-1.5 days^55^ (95% CI) and 2.01-5.61 days^56^ (95% CI), respectively, so subtracting one day for influenza and two days for omicron from the day of secondary case symptom onset would estimate the latest likely day of transmission. For example, all studies reported that symptom onset occurred in at least 80% of secondary cases by the end of day six post-primary case symptom onset for influenza and day seven for omicron (Figure 4), suggesting at least 80% of transmissions from a primary case would be estimated to have occurred by the end of day five for both pathogens. This is four days sooner than the resolution of shedding for 80% of participants with both pathogens on day nine, suggesting the duration of shedding is not the most accurate marker for the risk of transmission. These findings echo other studies that report a higher risk for transmission earlier in the course of illness for SARS-CoV-2.^39,57,58^

Work restrictions applied to potentially contagious HCWs in U.S. healthcare settings are typically longer than those applied to people in the community. In healthcare settings, decisions about the duration of work restriction for HCWs have historically relied primarily on viral shedding data despite their limitations. Ideally, the duration can be informed by observations of transmission collected in epidemiologic studies to give a more complete picture of transmission risk. Decisions about work restrictions will balance the benefits of reducing risk of transmission with potential unintended consequences of work restrictions like changes to staffing that could impact safe patient care or a safe work environment.^57^

Other infection prevention and control measures may shorten the duration of viral shedding or reduce the risk of transmission of influenza or omicron. Limited evidence suggests there may be a reduction in the duration of viral shedding in vaccinated adults who are mildly ill with omicron, and no evidence was retrieved on the effect of vaccination on influenza shedding, therefore the effect of vaccination status on shedding duration to inform decisions about duration of work restrictions is unclear.^58,59^ Additional studies may clarify this relationship but could vary by pathogen. For influenza, neuraminidase inhibitors have been shown to reduce viral shedding by 1-2 days and may reduce the risk for transmission to others.^60,61^ Although decreases in viral shedding have been reported for adults receiving Paxlovid for omicron infections, the risk for rebound viremia may negate potential for decreasing risk for transmission to others.^57,62,63^ Finally, masking of potentially contagious individuals for source control is a strategy that can reduce the risk for transmission to others in healthcare settings.^64^ However, the potential reduction in risk is difficult to quantify and may be imperfect because it depends on factors such as how well the mask fits and whether it is consistently and correctly worn at all times. This suggests that masking for source control is not to be relied upon to replace exclusion of contagious HCWs from work, particularly when HCWs are most contagious.

The strengths of this review include the use of multiple approaches to estimate the risk period for transmission of influenza and omicron and the visualization of this evidence, using rapid review techniques. This review examined evidence in previously healthy adults, and the findings might not apply to other populations (e.g., immunocompromised persons). The inclusion of shedding data measured from diagnosis rather than symptom onset may artificially lengthen the duration of shedding in some studies. It is challenging to identify when symptoms might be improving, which is subjective, making the relationship between resolution of viral shedding and symptoms difficult to ascertain. For the outcome of secondary case symptom onset, serial intervals of zero or less were excluded, which may have excluded valid transmission pairs where the incubation period of the primary case was longer than that of the secondary case, and may have resulted in an overestimation of the duration of contagiousness. Further, some influenza studies truncated reporting symptom onset in secondary cases on a specific day (transmissions occurring beyond that day were assumed to not be from that index case), which may also underestimate the serial interval.^41,42,46,50^ These limitations further underscore the conservative nature of these estimates and may increase confidence in the findings. This review did not assess recall bias and confounding due to population-level presence or absence of comorbidities. Finally, several influenza challenge studies from the 1970s & 1980s did not report data in a manner that was useable for the purposes of this review. It is possible that this excluded literature contained data that more extensively addressed viral shedding and symptom progression.

### Conclusions

This review summarizes the currently available data on viral shedding for influenza A and the omicron variant of SARS-CoV-2, its relationship to symptoms, and the timing of symptom onset in secondary cases to refine the duration of infectivity for HCWs. Cumulative serial interval data can be used to estimate the daily progression of risk for transmission from a symptomatic HCW with omicron or influenza. Ensuring the availability of pair-level serial interval data or reporting the daily cumulative proportion of symptom onset in secondary cases may provide a more direct measure of the risk of transmission for these respiratory viruses to, inform prevention interventions like work restrictions.

## Supporting information

Stone et al. supplementary material Stone et al. supplementary material

## Supplementary material

The supplementary material for this article can be found at https://doi.org/10.1017/ice.2025.11
