# Supplementary material for: Risk period for transmission of SARS-CoV-2 and seasonal influenza: a rapid review
Source: Infect Control Hosp Epidemiol. 2025 Jan 24;46(3):227–35. doi: 10.1017/ice.2025.11 (PMC11883656; doi:10.1017/ice.2025.11)
Supplement: Stone et al. supplementary material [file S0899823X2500011Xsup001.docx]

**Supplementary Material: Risk Period for Transmission of SARS-CoV-2 and Seasonal Influenza: A Rapid Review**

***Citation**

# **Part I: Supplemental Methods**

## **I.A Methods**

This rapid review is reported following the standards set by the Preferred Reporting Items for Systematic reviews and Meta-Analyses extension for Scoping Reviews (PRISMA-ScR) because the standard for rapid reviews is not finalized.^1^ Brief protocols were developed, but not published.

### **I.A.1 Key Questions**

The research questions developed by occupational health, infectious disease, and systematic review methodology subject matter experts are:

Among symptomatic adults infected with SARS-CoV-2 (Omicron Variant) and influenza A:

1. What is the duration of viral shedding measured from symptom onset or diagnosis using viral culture or RT-qPCR?
2. What is the association between the resolution of symptoms, specifically fever, and the resolution of viral shedding measured using culture, RT-qPCR, or RT-PCR?
3. What is the pair-level serial interval, defined as the number of days between symptom onset in primary and secondary cases?

### **I.A.2 Search Strategy & Study Selection**

A CDC information specialist developed search strategies for SARS-CoV-2 for each of the three research questions and adapted each strategy for influenza. The informationist performed these searches in MEDLINE, Embase, Cochrane Library, CINAHL, and Scopus from the start of each data to July 26 or August 1, 2024. The detailed search strategies are provided in [S*upplemental Tables s4-s9*.](#_Part_III.A_Search)

Results of the literature searches were uploaded into EndNote 21 (Clarivate Analytics©, Thomson Reuters, New York, NY, USA), duplicate records were removed, and unique titles and abstracts were uploaded to Covidence (Veritas Health Innovation Ltd., Melbourne, VIC, Australia) where a second round of deduplication was conducted. Two reviewers screened all titles and abstracts and removed irrelevant references. Titles and abstracts and subsequent full texts were screened by two reviewers and disagreements were resolved by consensus among reviewers. The reference lists of systematic reviews meeting inclusion criteria were mined for relevant studies. All studies were screened according to pre-identified exclusion criteria (Table s1), and the results of the study selection process for all six reviews are provided in [*Supplemental Figures s7-s12*](#_Part_III.B_PRISMA)

Studies were included if the full text was available and written in English; and if the study was relevant to the research question, reported primary data, and reviewers could verify complete methods (i.e., articles were not conference abstracts or posters.) Exclusion criteria were selected a priori to minimize anticipated risks of bias in the final body of evidence. Research question-specific exclusion criteria are listed in Table s1, according to domain of bias.

**Table s1:** Exclusion criteria and relevant rationale.

| **Exclusion criteria/ exclude studies** | **Domain of Bias** | **Rationale** |
| --- | --- | --- |
| RQ1, 2, & 3: Exclude studies conducted in populations that are predominantly children-based. | Confounding | Minimize or eliminate confounding or effect measure modification due to differences in age-related shedding & risk of transmission |
| RQ1, 2, & 3: Exclude studies conducted in populations that are highly comorbid or immunocompromised. | Confounding | Minimize or eliminate confounding or effect measure modification due to differences in shedding & risk of transmission due to comorbidities or immunocompromised status. |
| RQ1, 2, & 3: Exclude studies conducted in populations that are hospitalized for moderate, severe, or critical illness | Confounding | Minimize or eliminate confounding or effect measure modification due to differences in shedding & risk of transmission due to severity of illness |
| RQ1 & RQ2: Exclude studies that collect shedding data at any frequency other than daily. | Information Bias: measurement | Minimize or eliminate unclear duration of shedding. |
| - RQ1. Exclude studies reporting results collected using diagnostic tests that are not culture or RT-qPCR | Information Bias: measurement | Eliminate bias associated with comparing other test methods (e.g., antigen tests) to the gold standard of culture, or improved measurement of quantitative PCR |
| RQ1, 2, & 3: Exclude studies examining patients with non-omicron variants of SARS-CoV-2, non-influenza A types or subtypes, or parainfluenza | Information Bias: measurement | Minimize or eliminate measurement bias associated with longer serial intervals and durations of shedding associated with prior variants of SARS-CoV-2 and other types of influenza |
| RQ2. Exclude studies reporting results collected using diagnostic tests that are not culture, RT-qPCR, or RT-PCR | Information Bias: measurement | Eliminate bias associated with comparing other test methods (e.g., antigen tests), however due to concerns that many influenza studies were published prior to the widespread use of RT-qPCR, RT-PCR was included |
| RQ1 & 2. Exclude studies reporting RT-qPCR (omicron) or RT-PCR (influenza) results reported as CT values. | Information Bias: measurement | Eliminate bias that may be associated with comparing CT values and log_10_ copies per mL values across studies |
| RQ1 & 2. Exclude studies that do not report the end of shedding for <90% of the study population | Information bias: measurement | Eliminate biases associated with incomplete data and an unclear duration of shedding. |
| RQ 1& 2: Exclude studies that do measure shedding from symptom onset, diagnosis, or inoculation, or are unclear in their reporting of the start of measurement | Information bias: measurement | Eliminate biases associated with incomplete data and an unclear duration of shedding. |
| RQ2: Exclude studies that do not collect daily symptom data either in person, via telephone, or using a daily log. | Information bias: recall bias | Eliminate bias associated with mis-remembering past events. |
| RQ3. Exclude studies that do not report data, either in chart, table, supplement, or requested data that enables the determination of the day of symptom onset in the secondary case measured in days from symptom onset in the primary case. | Information bias: measurement | Eliminate bias associated with estimating the cumulative proportion of secondary case day of symptom onset using incomplete data or inappropriate data. |
| RQ3. Exclude studies that report on serial estimates based on indirect transmission. | Information bias: misclassification | Eliminate bias in the data from inaccurate measurements of serial interval |

### **I.A.3 Data Extraction**

Data was extracted by two reviewers using a standardized Microsoft Excel (2021) form, and differences were reconciled by discussion. Extracted data included study characteristics, population characteristics, the incubation period, and the outcomes of interest (Table s2). Outcome data were extracted as presented in the studies or calculated using data provided, abstracted from relevant figures using an online digitizing application ([PlotDigitizer Online App](https://plotdigitizer.com/app)).^2^ For RQ1, if a study used both culture and RT-qPCR for diagnostic testing, only culture data was extracted. For RQ3, serial intervals of zero, or when symptom onset in the infector and infectee occurred on the same day, were not extracted. For any research question, when interventional studies met inclusion criteria, only placebo group data was extracted. Potential areas for risk of bias were identified a priori to guide study selection, and possible confounding factors were identified for use in data analysis, thus risk of bias assessments of studies were not conducted.

Concerns for information bias (such as diagnostic test type) and selection bias were identified a priori and guided selection criteria (Table s1). Concerns for confounding were also identified a priori, such as concurrent infection prevention and control interventions such as masking and lockdown orders, vaccination status, differences in sub-variants or strains, and the timing of data collection in the spread of omicron for those studies. Due to this approach, a study-level risk of bias assessment was not conducted.

**Table s2:** Outcomes of interest for each research question measured in days.

| **Research Question** | Outcome of interest | Definition |
| --- | --- | --- |
| **RQ.1** | Duration of respiratory viral shedding measured by qPCR or viral culture and reported as log_10_ copies/mL or CT values. | The day, measured daily from symptom onset, that at least one diagnostic test (preferably two) returns a negative value. |
| **RQ.2** | Duration of respiratory viral shedding measured daily using RT-PCR, RT-qPCR, or culture (days) | The day, measured from symptom onset, at least one diagnostic test (preferably two) returns a negative value. |
| **RQ.2** | Duration of fever by clinician evaluation or self-reported via questionnaire. (days) | The day, measured daily, that the participants temperature returns to normal, or the fever “ends”. |
| **RQ.3** | Symptom onset in secondary case (day) | The day symptom onset in the secondary case occurs, as measured from the day of symptom onset in the primary case. May be self-reported |

### **I.A.4 Data Analysis**

Shedding and symptom outcomes were analyzed from symptom onset, diagnosis, or inoculation (for influenza challenge studies) in the participant. Serial interval outcomes were analyzed from symptom onset in the primary case, which was defined as day zero. The cumulative proportion of study participants with the outcomes of interest were calculated at the study-level. Data was analyzed using Microsoft Excel (2021) and R Studio. Analysis for risk stratification was conducted for study-level cumulative proportions of 70% - 100% participants experiencing resolution of shedding for individuals or symptom onset in secondary cases of pairs. Stratified analyses were performed to visually inspect the influence of confounding factors. These factors included household transmissions compared with community transmission, collectivist and individualist cultures to approximate masking, vaccination status, sub-variant and strain, pandemic and seasonal influenza, and non-pharmaceutical interventions (NPIs) such as masking and lockdown policies.

**Part II. Supplemental Figures**

**Figure s1.** Cumulative proportion (%) of omicron-infected participants with resolution of shedding stratified by vaccination status.


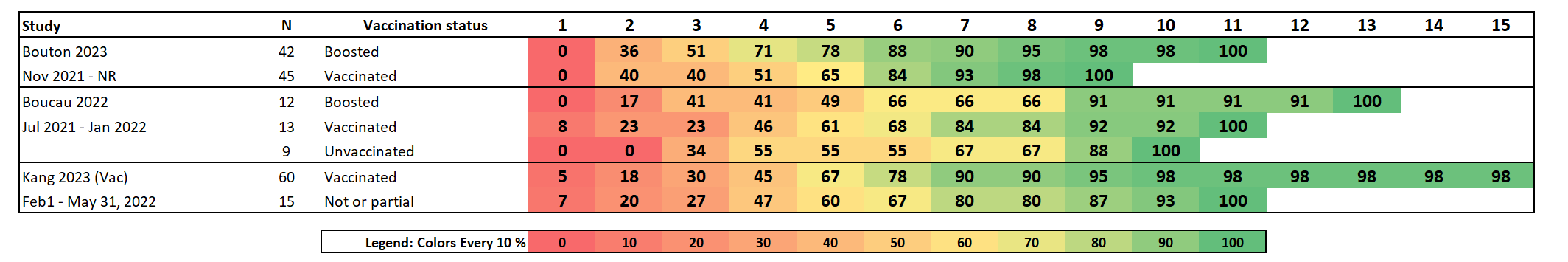


**Figure s2** Cumulative proportion (%) of omicron- or influenza-infected participants with shedding after resolution of symptoms, or both resolution of shedding and resolution of symptoms.


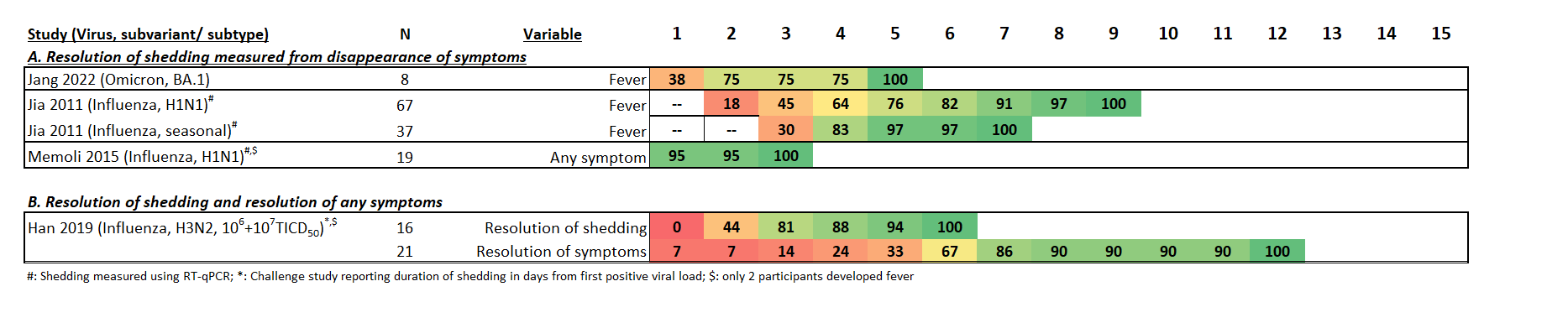


**Figure s3** Cumulative proportion (%) of symptom onset in secondary omicron (A) or influenza (B) cases stratified by collectivist or individualist societies to approximate mask wearing.

**
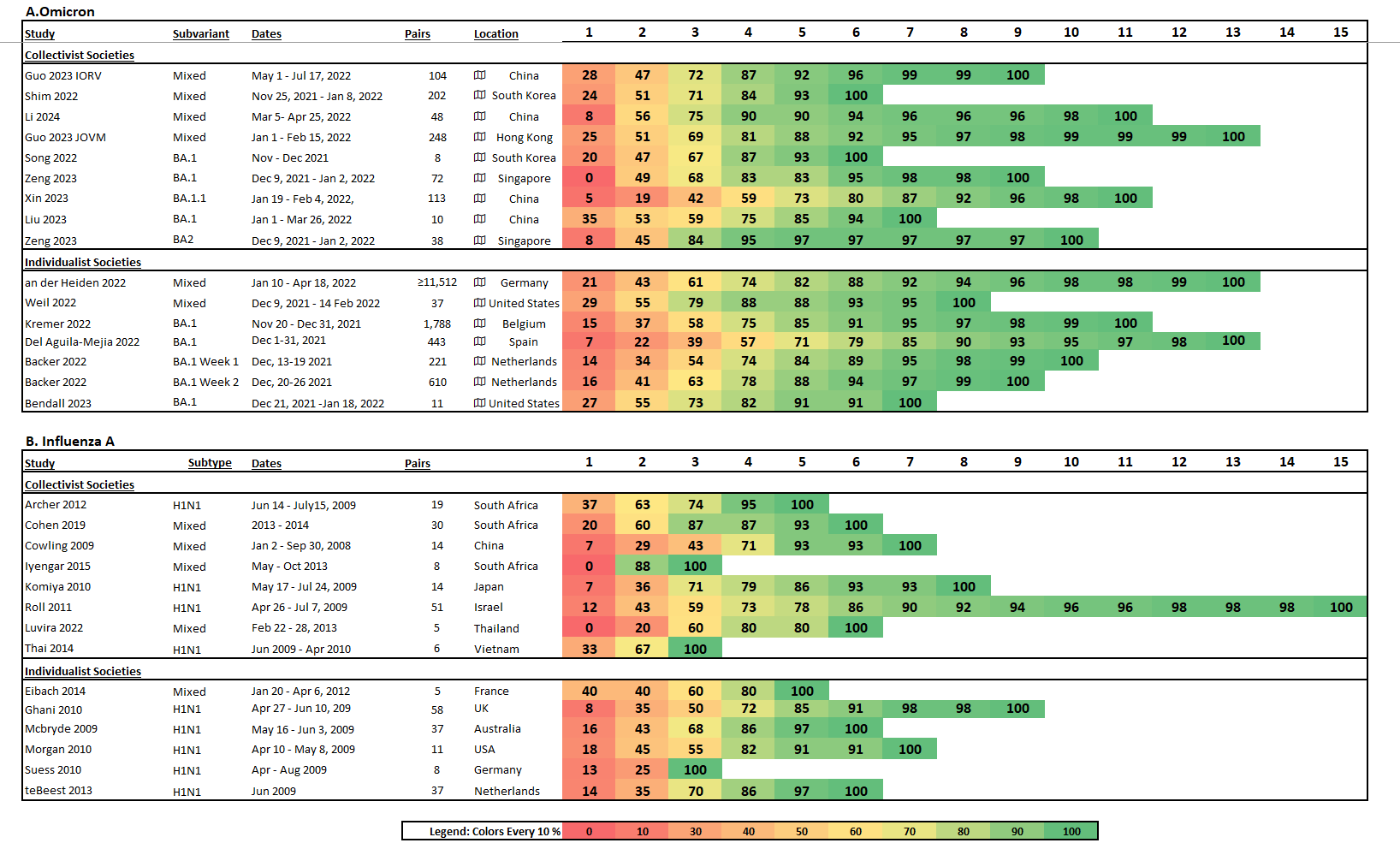
**

**Figure s4** Cumulative proportion (%) of symptom onset in secondary omicron (A) or influenza (B) cases stratified by data collection in households, household + non-household settings, and household and other settings.

**
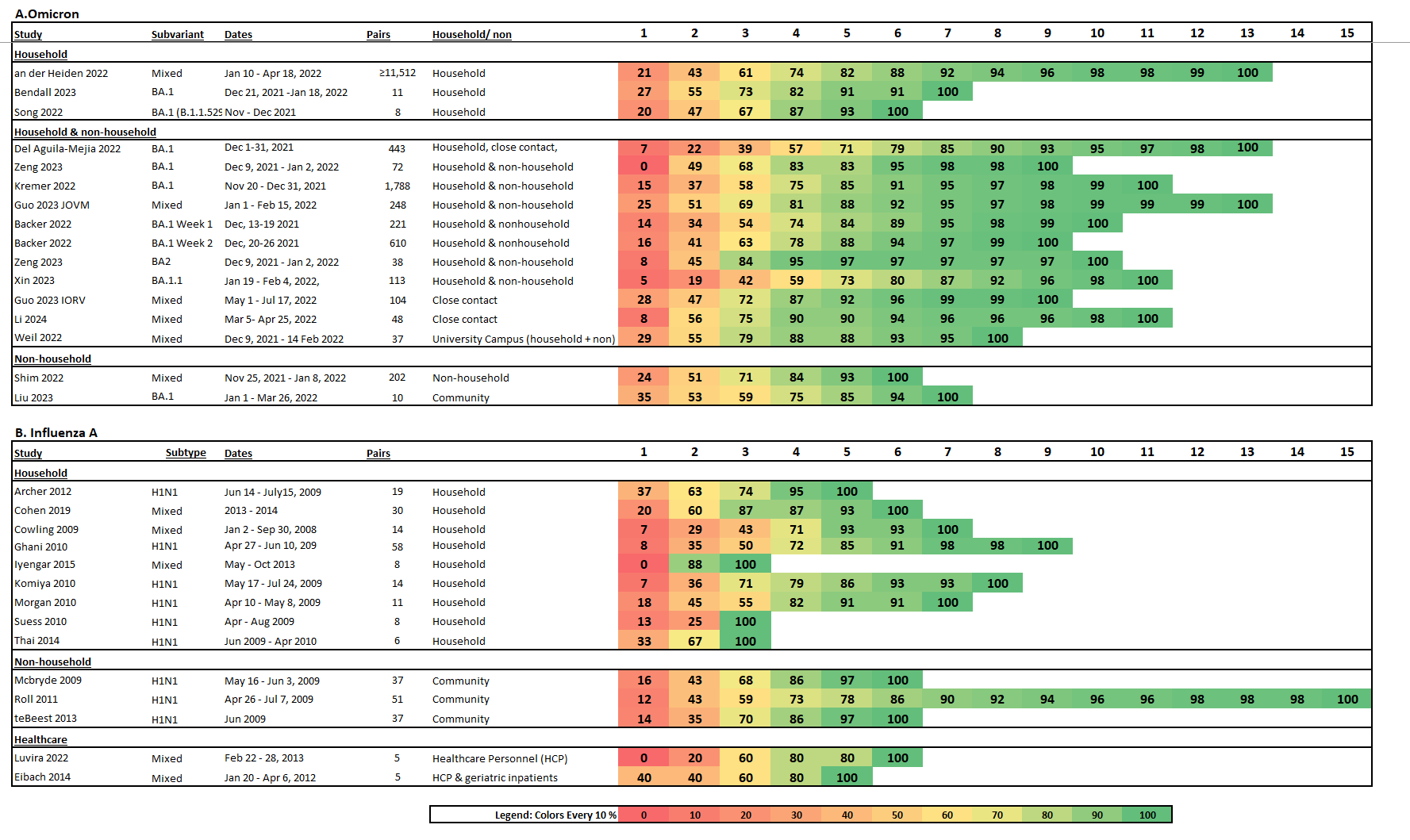
**

**Figure s5** Cumulative proportion (%) of symptom onset in secondary omicron cases stratified by the proportion of vaccinations in the population at that time.


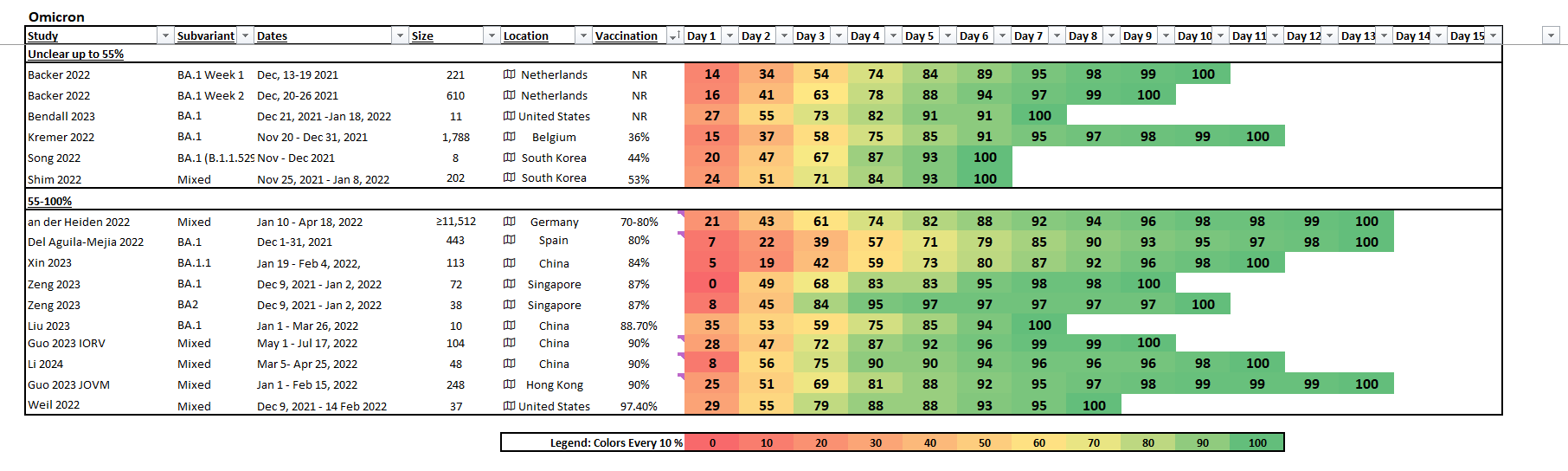


**Figure s6** Cumulative proportion (%) of symptom onset in secondary omicron cases stratified by the implementation of societal non-pharmaceutical interventions (NPI) at that time.

**
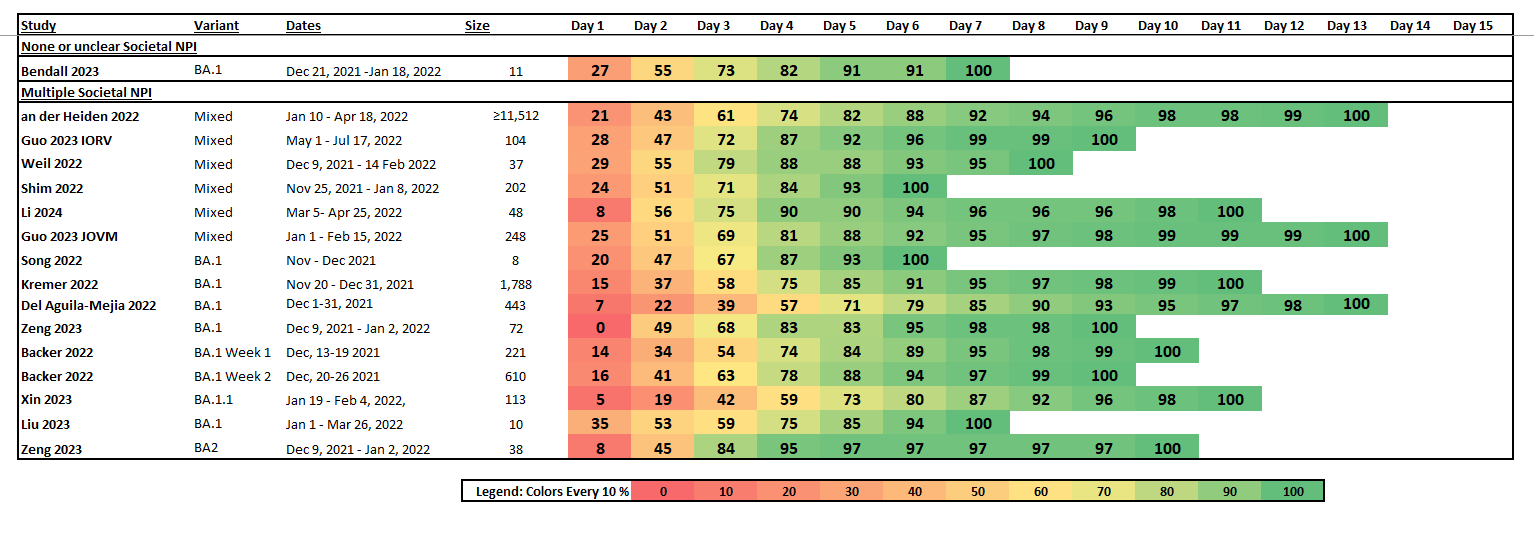
**

# **Part III: Extracted Data**

**Table s3:** Extracted data from included studies.

| **Study** | **RQRQ** | **N** | **Population** | **Location** | **Dates** | **Pathogen** | **Strain/ Variant** | **Diagnostic test** | **Measured since** | **Vaccination** | **Household transmission** | **Societal NPIs** | **Figure extraction** |
| --- | --- | --- | --- | --- | --- | --- | --- | --- | --- | --- | --- | --- | --- |
| an der Heiden 2022^3^ | RQ3 | 11,512 | Household | Germany | Oct 2020 – May 24, 2022 | SARS-CoV-2 | BA.2, BA.4, & BA.5 | NR | SO1C | NR | Y | NR | F-D |
| Archer 2012^4^ | RQ3 | 19 | Household | South Africa | Jun 14 – Jul 15, 2009 | Influenza | H1N1 | PCR | SO1C | NR | Y | NR | F |
| Backer 2022^5^ | RQ3 | 221 | Community | Netherlands | Dec 13 – 26, 2021 | SARS-CoV-2 | BA.1 | RT-PCR | SO1C | NR | Y | Y | R |
| Bendall 2023^6^ | RQ3 | 11 | Household | USA | Nov 18, 2020 – Jan 19, 2022 | SARS-CoV-2 | BA.1 | RT-qPCR | SO1C | NR | Y | NR | R |
| Boucau 2022^7^ | RQ1 | 34 | Outpatients | USA | Jul 2021 – Jan 2022 | SARS-CoV-2 | BA.1 | Culture | SO or diagnosis | 73% | NA | NA | F-D |
| Bouton 2023^8^ | RQ1 | 75 | Students & staff | USA | Nov 2021 - NR | SARS-CoV-2 | NR | Culture | SO or diagnosis | 100% | NA | NA | F-D |
| Cohen 2019^9^ | RQ3 | 30 | Household | South Africa | 2013 - 2014 | Influenza | H3N2, H1N1, A, B | RT-PCR | SO1C | 1 – 4% | Y | NR | F |
| Cowling 2009^10^ | RQ3 | 14 | Household | Hong Kong, China | 2007 | Influenza | NR | RT-PCR | SO1C | NR | Y | NR | R |
| Del Aguila-Mejia 2022^11^ | RQ3 | 443 | Community | Spain | Nov – Dec 2021 | SARS-CoV-2 | BA.1 | PCR | SO1C | NR | Y | NR | F-D |
| Doyle 1998^12^ | RQ1 | 42 | Adult volunteers | USA | NR | Influenza | H1N1 | Culture | Inoculation | NR | NA | NA | F-D |
| Eibach 2014^13^ | RQ3 | 5 | HCP and geriatric inpatients | France | Jan 20 – Apr 6, 2012 | Influenza | H3N2 | PCR | SO1C | 33% HCP,  67% patients | N | NR | F |
| Ghani 2010^14^ | RQ3 | 58 | Household | UK | Apr 27 – Jun 10, 2009 | Influenza | H1N1 | NR | SO1C | NR | Y | NR | F-D |
| Guo 2023 (IORV)^15^ | RQ3 | 104 | Community | China | May 1 – Jul 17, 2022 | SARS-CoV-2 | BA.2, BA.4, & BA.5 | NR | SO1C | NR | NR | NR | R |
| Guo 2023^16^ (JOMV) | RQ3 | 248 | Community | Hong Kong, China | Jan 1 – Feb 15, 2022 | SARS-CoV-2 | BA.1 & BA.2 | RT-PCR | SO1C | NR | Y | NR | F-D |
| Han 2019^17^ | RQ1, RQ2 | 37 | Adult volunteers | USA | Dec 2015 – July 2017 | Influenza | H3N2 | Culture | SO | N | NA | NA | R |
| Hooker 2021^18^ | RQ1 | 12 | Adult volunteers | USA | Jun – Jul 1997 | Influenza | H1N1 | Culture | Inoculation | NR | NA | NA | R |
| Iyengar 2015^19^ | RQ3 | 8 | Household | South Africa | May – Oct 2013 | Influenza | H1N1, H3N2, B | PCR | SO1C | 3 – 5% | Y | NR | F |
| Jang 2022^20^ | RQ1, RQ2 | 9 | Community | South Korea | December 2021 | SARS-CoV-2 | NR | Culture | SO | 0% | NA | NA | F |
| Jia 2011^21^ | RQ2 | 104 | Adult patients | China | Sept 2009 – Jan 2010 | Influenza | H1N1, seasonal | RT-PCR | SO or defervescence | NR | NA | NA | R |
| Jung 2023^22^ | RQ1 | 32 | HCP | South Korea | Mar 14 – Apr 3, 2022 | SARS-CoV-2 | BA.1 & BA.2 | Culture | SO | 100% | NA | NA | R |
| Kang 2023 (JOMV)^23^  Related: Kang 2023^24^ (Vac) | RQ1 | 34 | Patients | South Korea | Feb 1, 2021 – May 31, 2022 | SARS-CoV-2 | NR | Culture | SO | 70%; Stratified analysis in Kang 2023 (Vac) | NA | NA | F-D |
| Kang 2023^25^ (JOI) | RQ1 | 82 | HCP | South Korea | Jan 14 – Aug 2, 2022 | SARS-CoV-2 | BA.1, BA.2, & BA.5 | Culture | SO or diagnosis | 100% | NA | NA | R |
| Killingley 2010^26^ | RQ1 | 4 | Adult patients | UK | Sept 14, 2009 – Jan 25, 2010 | Influenza | H1N1 | Culture | SO | N | NA | NA | F |
| Killingley 2016^27^ | RQ1 | 27 | Adult patients | UK | Sept 2009 – Jan 2011 | Influenza | H1N1 | Culture | SO | NR | NA | NA | F-D |
| Komiya 2010^28^ | RQ3 | 14 | Household | Japan | May 17 – Jul 24, 2009 | Influenza | H1N1 | RT-PCR | SO1C | NR | Y | Y | F |
| Kremer 2022^29^ | RQ3 | 2,161 | Community | Belgium | Nov 19 – Dec 31, 2021 | SARS-CoV-2 | BA.1 | PCR | SO1C | NR | Y | NR | R |
| Li 2024^30^ | RQ3 | 48 | Community | China | Mar 5 – Apr 25, 2024 | SARS-CoV-2 | NR | RT-PCR | SO1C | NR | NR | Y | R |
| Liu 2023^31^ | RQ3 | 546 | Community | China | Jan 1 – Mar 26, 2022 | SARS-CoV-2 | BA.1 & BA.2 | NR | SO1C | 88.7% | NR | Y | F-D |
| Luvira 2022^32^ | RQ3 | 5 | HCP | Thailand | Feb 22 – 28, 2013 | Influenza | H3N2, B | RT-PCR | SO1C | 50% | N | NR | F |
| McBryde 2009^33^ | RQ3 | 37 | Community | Australia | May 16 – Jun 3, 2009 | Influenza | H1N1 | NR | SO1C | NR | Y | NR | F |
| Memoli 2015^34^ | RQ1, RQ2 | 20 | Adult volunteers | USA | Apr 2012 – Jun 2013 | Influenza | H1N1 | RT-qPCR | Inoculation | N | NA | NA | F |
| Morgan 2010^35^ | RQ3 | 11 | Household | USA | Apr 10 – May 8, 2009 | Influenza | H1N1 | RT-PCR | SO1C | 29% | Y | NR | R |
| Roll 2011^36^ | RQ3 | 51 | Community | Israel | Apr 26 – Jul 7, 2009 | Influenza | H1N1 | PCR | SO1C | NR | Y | NR | F |
| Shim 2022^37^ | RQ3 | 202 | Community | South Korea | Nov 25, 2012 – Jan 8, 2022 | SARS-CoV-2 | BA.1 & BA.2 | NR | SO1C | 58% | NR | NR | F-D |
| Smith-Jeffcoat 2024^38^ | RQ1 | 236 | Community | USA | Nov 2022 – May 2023 | SARS-CoV-2 | NR | Culture | SO or diagnosis | 40% | NA | NA | F-D |
| Song 2022^39^ | RQ3 | 12 | Community | South Korea | Nov 22 – Dec 15, 2021 | SARS-CoV-2 | BA.1 | RT-PCR | SO1C | 44% | Y | NR | R |
| Suess 2010^40^ | RQ3 | 8 | Household | Germany | Apr – Aug 2009 | Influenza | H1N1 | RT-PCR | SO1C | NR | Y | NR | R |
| teBeest 2013^41^ | RQ3 | 37 | Community | Netherlands | Jun 2009 | Influenza | H1N1 | PCR | SO1C | NR | Y | NR | R |
| Thai 2014^42^ | RQ3 | 6 | Household | Vietnam | Jun 2009 – Apr 2010 | Influenza | H1N1 | RT-PCR | SO1C | 0% | Y | NR | R |
| Weil 2022^43^ | RQ3 | 37 | Students | USA | Sept 10, 2021 – Feb 14, 2022 | SARS-CoV-2 | BA.1 & BA.2 | RT-qPCR | SO1C | 97.4% | Y | Y | F |
| Xin 2023^44^ | RQ3 | 113 | Community | China | Jan 23 – Feb 4, 2022 | SARS-CoV-2 | BA.1.1 | RT-PR | SO1C | 84.2% | Y | Y | F |
| Zeng 2023^45^ | RQ3 | 114 | Community | Singapore | Dec 9 - NR | SARS-CoV-2 | BA.1 & BA.2 | PCR | SO1C | 87% | Y | NR | F |
| RQ: Research Question; NR: Not reported in study; SO: Symptom onset; SO1C: Symptom onset in primary case; R: Data were reported in study; F: data extracted from figure; F-D: data was digitized from figure. | | | | | | | | | | | | | |

# **References**

1. Equator Network. Reporting guidelines under development for systematic reviews. 2024 [cited 2024 October 4]; Available from: <https://www.equator-network.org/library/reporting-guidelines-under-development/reporting-guidelines-under-development-for-systematic-reviews/#51>

2. Tierney JF, Stewart LA, Ghersi D, Burdett S, Sydes MR. Practical methods for incorporating summary time-to-event data into meta-analysis. Trials 2007;8(1):16.

3. An der Heiden M, Buchholz U. Serial interval in households infected with SARS-CoV-2 variant B.1.1.529 (Omicron) is even shorter compared to Delta. Epidemiol Infect 2022;150:e165.

4. Archer BN, Timothy GA, Cohen C, Tempia S, Huma M, Blumberg L, et al. Introduction of 2009 pandemic influenza A virus subtype H1N1 into South Africa: clinical presentation, epidemiology, and transmissibility of the first 100 cases. J Infect Dis 2012;206 Suppl 1(Suppl 1):S148-53.

5. Backer JA, Eggink D, Andeweg SP, Veldhuijzen IK, van Maarseveen N, Vermaas K, et al. Shorter serial intervals in SARS-CoV-2 cases with Omicron BA.1 variant compared with Delta variant, the Netherlands, 13 to 26 December 2021. Euro Surveill 2022;27(6).

6. Bendall EE, Callear AP, Getz A, Goforth K, Edwards D, Monto AS, et al. Rapid transmission and tight bottlenecks constrain the evolution of highly transmissible SARS-CoV-2 variants. Nat Commun 2023;14(1):272.

7. Boucau J, Marino C, Regan J, Uddin R, Choudhary MC, Flynn JP, et al. Duration of Shedding of Culturable Virus in SARS-CoV-2 Omicron (BA.1) Infection. N Engl J Med 2022;387(3):275-277.

8. Bouton TC, Atarere J, Turcinovic J, Seitz S, Sher-Jan C, Gilbert M, et al. Viral Dynamics of Omicron and Delta Severe Acute Respiratory Syndrome Coronavirus 2 (SARS-CoV-2) Variants With Implications for Timing of Release from Isolation: A Longitudinal Cohort Study. Clin Infect Dis 2023;76(3):e227-e233.

9. Cohen C, Tshangela A, Valley-Omar Z, Iyengar P, Von Mollendorf C, Walaza S, et al. Household Transmission of Seasonal Influenza From HIV-Infected and HIV-Uninfected Individuals in South Africa, 2013-2014. J Infect Dis 2019;219(10):1605-1615.

10. Cowling BJ, Fang VJ, Riley S, Malik Peiris JS, Leung GM. Estimation of the serial interval of influenza. Epidemiology 2009;20(3):344-7.

11. Del Águila-Mejía J, Wallmann R, Calvo-Montes J, Rodríguez-Lozano J, Valle-Madrazo T, Aginagalde-Llorente A. Secondary Attack Rate, Transmission and Incubation Periods, and Serial Interval of SARS-CoV-2 Omicron Variant, Spain. Emerg Infect Dis 2022;28(6):1224-1228.

12. Doyle WJ, Skoner DP, Alper CM, Allen G, Moody SA, Seroky JT, et al. Effect of rimantadine treatment on clinical manifestations and otologic complications in adults experimentally infected with influenza A (H1N1) virus. J Infect Dis 1998;177(5):1260-5.

13. Eibach D, Casalegno JS, Bouscambert M, Bénet T, Regis C, Comte B, et al. Routes of transmission during a nosocomial influenza A(H3N2) outbreak among geriatric patients and healthcare workers. J Hosp Infect 2014;86(3):188-93.

14. Ghani A, Baguelin M, Griffin J, Flasche S, van Hoek AJ, Cauchemez S, et al. The Early Transmission Dynamics of H1N1pdm Influenza in the United Kingdom. PLoS Curr 2009;1:Rrn1130.

15. Guo Z, Zhao S, Yam CHK, Li C, Jiang X, Chow TY, et al. Estimating the serial intervals of SARS-CoV-2 Omicron BA.4, BA.5, and BA.2.12.1 variants in Hong Kong. Influenza Other Respir Viruses 2023;17(2):e13105.

16. Guo Z, Zhao S, Mok CKP, So RTY, Yam CHK, Chow TY, et al. Comparing the incubation period, serial interval, and infectiousness profile between SARS-CoV-2 Omicron and Delta variants. J Med Virol 2023;95(3):e28648.

17. Han A, Czajkowski LM, Donaldson A, Baus HA, Reed SM, Athota RS, et al. A Dose-finding Study of a Wild-type Influenza A(H3N2) Virus in a Healthy Volunteer Human Challenge Model. Clin Infect Dis 2019;69(12):2082-2090.

18. Hooker KL, Ganusov VV. Impact of Oseltamivir Treatment on Influenza A and B Virus Dynamics in Human Volunteers. Front Microbiol 2021;12:631211.

19. Iyengar P, von Mollendorf C, Tempia S, Moerdyk A, Valley-Omar Z, Hellferscee O, et al. Case-ascertained study of household transmission of seasonal influenza - South Africa, 2013. J Infect 2015;71(5):578-86.

20. Jang YR, Kim JM, Rhee JE, Kim D, Lee NJ, Lee H, et al. Clinical Features and Duration of Viral Shedding in Individuals With SARS-CoV-2 Omicron Variant Infection. Open Forum Infect Dis 2022;9(7):ofac237.

21. Jia N, Gao Y, Suo JJ, Xie LJ, Yan ZQ, Xing YB, et al. Viral shedding in Chinese young adults with mild 2009 H1N1 influenza. Chin Med J (Engl) 2011;124(10):1576-9.

22. Jung J, Kang SW, Lee S, Park H, Kim JY, Kim SK, et al. Risk of transmission of COVID-19 from healthcare workers returning to work after a 5-day isolation, and kinetics of shedding of viable SARS-CoV-2 variant B.1.1.529 (Omicron). J Hosp Infect 2023;131:228-233.

23. Kang SW, Kim JY, Park H, Lim SY, Kim J, Chang E, et al. Comparison of secondary attack rate and viable virus shedding between patients with SARS-CoV-2 Delta and Omicron variants: A prospective cohort study. J Med Virol 2023;95(1):e28369.

24. Kang SW, Park H, Yeun Kim J, Bae JY, Park MS, Kim SH. Comparison of culture-competent virus shedding duration of SARS-CoV-2 Omicron variant in regard to vaccination status: A prospective cohort study. Vaccine 2023;41(17):2769-2772.

25. Kang SW, Park H, Kim JY, Lim SY, Lee S, Bae JY, et al. Comparison of the clinical and virological characteristics of SARS-CoV-2 Omicron BA.1/BA.2 and omicron BA.5 variants: A prospective cohort study. J Infect 2023;86(5):e148-e151.

26. Killingley B, Greatorex J, Cauchemez S, Enstone JE, Curran M, Read RC, et al. Virus shedding and environmental deposition of novel A (H1N1) pandemic influenza virus: interim findings. Health Technol Assess 2010;14(46):237-354.

27. Killingley B, Greatorex J, Digard P, Wise H, Garcia F, Varsani H, et al. The environmental deposition of influenza virus from patients infected with influenza A(H1N1)pdm09: Implications for infection prevention and control. J Infect Public Health 2016;9(3):278-88.

28. Komiya N, Gu Y, Kamiya H, Yahata Y, Yasui Y, Taniguchi K, et al. Household transmission of pandemic 2009 influenza A (H1N1) virus in Osaka, Japan in May 2009. J Infect 2010;61(4):284-8.

29. Kremer C, Braeye T, Proesmans K, André E, Torneri A, Hens N. Serial Intervals for SARS-CoV-2 Omicron and Delta Variants, Belgium, November 19-December 31, 2021. Emerg Infect Dis 2022;28(8):1699-1702.

30. Li P, Wen L, Sun B, Sun W, Chen H. Retrospective estimation of the time-varying effective reproduction number for a COVID-19 outbreak in Shenyang, China: An observational study. Medicine (Baltimore) 2024;103(22):e38373.

31. Liu C, Lu J, Li P, Feng S, Guo Y, Li K, et al. A comparative study on epidemiological characteristics, transmissibility, and pathogenicity of three COVID-19 outbreaks caused by different variants. Int J Infect Dis 2023;134:78-87.

32. Luvira V, Thippornchai N, Leaungwutiwong P, Siripoon T, Piroonamornpun P, Phumratanaprapin W, et al. Evidence of transmission of influenza A and influenza B co-infection in healthcare workers. J Infect Dev Ctries 2022;16(7):1199-1205.

33. McBryde E, Bergeri I, van Gemert C, Rotty J, Headley E, Simpson K, et al. Early transmission characteristics of influenza A(H1N1)v in Australia: Victorian state, 16 May - 3 June 2009. Euro Surveill 2009;14(42).

34. Memoli MJ, Czajkowski L, Reed S, Athota R, Bristol T, Proudfoot K, et al. Validation of the wild-type influenza A human challenge model H1N1pdMIST: an A(H1N1)pdm09 dose-finding investigational new drug study. Clin Infect Dis 2015;60(5):693-702.

35. Morgan OW, Parks S, Shim T, Blevins PA, Lucas PM, Sanchez R, et al. Household transmission of pandemic (H1N1) 2009, San Antonio, Texas, USA, April-May 2009. Emerg Infect Dis 2010;16(4):631-7.

36. Roll U, Yaari R, Katriel G, Barnea O, Stone L, Mendelson E, et al. Onset of a pandemic: characterizing the initial phase of the swine flu (H1N1) epidemic in Israel. BMC Infect Dis 2011;11:92.

37. Shim E, Choi W, Kwon D, Kim T, Song Y. Transmission Potential of the Omicron Variant of Severe Acute Respiratory Syndrome Coronavirus 2 in South Korea, 25 November 2021-8 January 2022. Open Forum Infect Dis 2022;9(7):ofac248.

38. Smith-Jeffcoat SE, Mellis AM, Grijalva CG, Talbot HK, Schmitz J, Lutrick K, et al. SARS-CoV-2 Viral Shedding and Rapid Antigen Test Performance - Respiratory Virus Transmission Network, November 2022-May 2023. MMWR Morb Mortal Wkly Rep 2024;73(16):365-371.

39. Song JS, Lee J, Kim M, Jeong HS, Kim MS, Kim SG, et al. Serial Intervals and Household Transmission of SARS-CoV-2 Omicron Variant, South Korea, 2021. Emerg Infect Dis 2022;28(3):756-759.

40. Suess T, Buchholz U, Dupke S, Grunow R, an der Heiden M, Heider A, et al. Shedding and transmission of novel influenza virus A/H1N1 infection in households--Germany, 2009. Am J Epidemiol 2010;171(11):1157-64.

41. te Beest DE, Wallinga J, Donker T, van Boven M. Estimating the generation interval of influenza A (H1N1) in a range of social settings. Epidemiology 2013;24(2):244-50.

42. Thai PQ, Mai le Q, Welkers MR, Hang Nle K, Thanh le T, Dung VT, et al. Pandemic H1N1 virus transmission and shedding dynamics in index case households of a prospective Vietnamese cohort. J Infect 2014;68(6):581-90.

43. Weil AA, Luiten KG, Casto AM, Bennett JC, O'Hanlon J, Han PD, et al. Genomic surveillance of SARS-CoV-2 Omicron variants on a university campus. Nat Commun 2022;13(1):5240.

44. Xin H, Wang Z, Feng S, Sun Z, Yu L, Cowling BJ, et al. Transmission dynamics of SARS-CoV-2 Omicron variant infections in Hangzhou, Zhejiang, China, January-February 2022. Int J Infect Dis 2023;126:132-135.

45. Zeng K, Santhya S, Soong A, Malhotra N, Pushparajah D, Thoon KC, et al. Serial Intervals and Incubation Periods of SARS-CoV-2 Omicron and Delta Variants, Singapore. Emerg Infect Dis 2023;29(4):814-817.

# **Part IV Search Strategies and Results**

## **Part IV.A Search Strategies**

**Table s4.** Search Strategies for RQ1.a: Omicron Shedding, Run August 1, 2024

| **Database** | **Strategy** |
| --- | --- |
| **Medline**  **(OVID)**  **1946-** | 1. Virus Shedding/ 2. (((viral OR virus) ADJ2 shed*) OR infectiousness OR infectivity OR contagiousness OR ((viral or virus) ADJ5 (emission* or emit*)) OR ((duration OR days OR time) ADJ5 shed*) OR (shed* ADJ5 (replica OR competent OR viable OR infecti*))).ti,ab,kf,hw. 3. 1 OR 2 4. exp Polymerase Chain Reaction/ OR exp clinical laboratory techniques/ 5. (polymerase chain reaction OR PCR OR RT-PCR OR RT-qPCR OR qPCR OR swab* OR nasal OR nasopharyngeal OR specimen* OR sample* OR test* OR assay* OR clinical technique* OR lab* OR cultures OR detect*).ti,ab,kf,hw. 6. 4 OR 5 7. 3 AND 6 8. Limit 7 to COVID-19 |
| **Embase**  **(OVID)**  **1974-** | 1. Virus Shedding/ 2. (((viral OR virus) ADJ2 shed*) OR infectiousness OR infectivity OR contagiousness OR ((viral or virus) ADJ5 (emission* or emit*)) OR ((duration OR days OR time) ADJ5 shed*) OR (shed* ADJ5 (replica OR competent OR viable OR infecti*))).ti,ab,kf,hw. 3. 1 OR 2 4. exp Polymerase Chain Reaction/ OR exp laboratory technique/ 5. (polymerase chain reaction OR PCR OR RT-PCR OR RT-qPCR OR qPCR OR swab* OR nasal OR nasopharyngeal OR specimen* OR sample* OR test* OR assay* OR clinical technique* OR lab* OR cultures OR detect*).ti,ab,kf,hw. 6. 4 OR 5 7. 3 AND 6 8. Limit 7 to COVID-19 9. Limit 8 to "pubmed/medline" |
| **Cochrane Library** | #1 [mh "Virus Shedding"]  #2 (((viral OR virus) NEAR/2 shed*) OR infectiousness OR infectivity OR contagiousness OR ((viral or virus) NEAR/5 (emission* or emit*)) OR ((duration OR days OR time) NEAR/5 shed*) OR (shed* NEAR/5 (replica OR competent OR viable OR infecti*))):ti,ab,kw  #3 #1 OR #2  #4 [mh ^"Polymerase Chain Reaction"] OR [mh ^"clinical laboratory techniques"]  #5 ("polymerase chain reaction" OR PCR OR "RT-PCR" OR "RT-qPCR" OR qPCR OR swab* OR nasal OR nasopharyngeal OR specimen* OR sample* OR test* OR assay* OR "clinical technique" OR "clinical techniques" OR lab* OR cultures OR detect*):ti,ab,kw  #6 #4 OR #5  #7 #3 AND #6  #8 [mh "Covid-19"] OR (coronavir* OR "corona virus" OR covid19 OR covid OR nCoV OR "novel CoV" OR "CoV 2" OR CoV2 OR sarscov2 OR sars2 OR 2019nCoV OR NCOV19):ti,ab,kw  #9 #7 AND #8 |
| **CINAHL**  **(EBSCOHost)** | S1 (MH "Virus Shedding")  S2 (((viral OR virus) N2 shed*) OR infectiousness OR infectivity OR contagiousness OR ((viral or virus) N5 (emission* or emit*)) OR ((duration OR days OR time) N5 shed*) OR (shed* N5 (replica OR competent OR viable OR infecti*)))  S3 S1 OR S2  S4 (MH "Polymerase Chain Reaction+") OR (MH "clinical laboratory techniques+")  S5 ("polymerase chain reaction" OR PCR OR "RT-PCR" OR "RT-qPCR" OR qPCR OR swab* OR nasal OR nasopharyngeal OR specimen* OR sample* OR test* OR assay* OR "clinical technique" OR "clinical techniques" OR lab* OR cultures OR detect*)  S6 S4 OR S5  S7 S3 AND S6  S8 (MH "COVID-19+") OR (TI (coronavir* OR "corona virus*" OR covid19 OR covid OR nCoV OR "novel CoV" OR "CoV 2" OR CoV2 OR sarscov2 OR sars2 OR 2019nCoV OR NCOV19)) OR (AB (coronavir* OR "corona virus*" OR covid19 OR covid OR nCoV OR "novel CoV" OR "CoV 2" OR CoV2 OR sarscov2 OR sars2 OR 2019nCoV OR NCOV19))  S9 S7 AND S8  **Limiters** - Exclude MEDLINE records |
| **Scopus** | (INDEXTERMS("Virus Shedding") OR TITLE-ABS-KEY(((viral OR virus) W/2 shed*) OR infectiousness OR infectivity OR contagiousness OR ((viral or virus) W/5 (emission* or emit*)) OR ((duration OR days OR time) W/5 shed*) OR (shed* W/5 (replica OR competent OR viable OR infecti*)))) AND (INDEXTERMS(("Polymerase Chain Reaction") OR ("laboratory techniques")) AND TITLE-ABS-KEY("polymerase chain reaction" OR PCR OR "RT-PCR" OR "RT-qPCR" OR qPCR OR swab* OR nasal OR nasopharyngeal OR specimen* OR sample* OR test* OR assay* OR "clinical technique" OR "clinical techniques" OR lab* OR cultures OR detect*)) AND (INDEXTERMS(COVID-19) OR TITLE-ABS-KEY(coronavir* OR "corona virus*" OR covid19 OR covid OR nCoV OR "novel CoV" OR "CoV 2" OR CoV2 OR sarscov2 OR sars2 OR 2019nCoV OR NCOV19)) AND NOT INDEX(medline) |

**Table s5.** Search Strategies for RQ1.b: Influenza Shedding, Run August 1, 2024

| **Database** | **Strategy** |
| --- | --- |
| **Medline**  **(OVID)**  **1946-** | 1. exp Influenza, Human/ 2. (influenza* OR flu).ti,ab,kf,hw. 3. 1 OR 2 4. exp virus shedding/ 5. ((shed* OR replica* OR competent OR infectiousness OR infectivity OR contagiousness emission* or emit* OR viable OR viability) ADJ5 (duration OR period OR time OR timing OR cease OR cessation)).ti,ab,kf,hw. 6. 4 OR 5 7. 3 AND 6 8. Limit 10 to English language |
| **Embase**  **(OVID)**  **1974-** | 1. exp Influenza/ 2. (influenza* OR flu).ti,ab,kf,hw. 3. 1 OR 2 4. exp virus shedding/ 5. ((shed* OR replica* OR competent OR infectiousness OR infectivity OR contagiousness emission* or emit* OR viable OR viability) ADJ5 (duration OR period OR time OR timing OR cease OR cessation)).ti,ab,kf,hw. 6. 4 OR 5 7. 3 AND 6 8. limit 7 to "pubmed/medline" 9. 7 NOT 8 10. limit 9 to conference abstract status 11. 9 NOT 10 12. Limit 11 to English language |
| **Cochrane Library** | 1. [mh "Influenza, Human"] 2. (influenza* OR flu):ti,ab,kw 3. #1 OR #2 4. [mh ^"virus shedding"] 5. ((shed* OR replica* OR competent OR infectiousness OR infectivity OR contagiousness OR emission* or emit* OR viable OR viability) NEAR/5 (duration OR period OR time OR timing OR cease OR cessation)):ti,ab,kw 6. #4 OR #5 7. #3 AND #6 |
| **CINAHL**  **(EBSCOHost)** | 1. (MH "Influenza, Human") 2. (influenza* OR flu) 3. S1 OR S2 4. (MH "virus shedding") 5. ((shed* OR replica* OR competent OR infectiousness OR infectivity OR contagiousness OR emission* or emit* OR viable OR viability) N5 (duration OR period OR time OR timing OR cease OR cessation)) 6. S4 OR S5 7. S3 AND S6   **Limiters** - Exclude MEDLINE records |
| **Scopus** | TITLE-ABS-KEY(influenza* OR flu) AND TITLE-ABS-KEY((shed* OR replica* OR competent OR infectiousness OR infectivity OR contagiousness OR emission* or emit* OR viable OR viability) W/5 (duration OR period OR time OR timing OR cease OR cessation)) AND NOT INDEX(medline) |

**Table s6.** Search Strategies for RQ2.a: Omicron Shedding & Symptom Association, Run July 26, 2024

| **Database** | **Strategy** |
| --- | --- |
| **Medline**  **(OVID)**  **1946-** | 1. Virus Shedding/ 2. (((viral OR virus) ADJ2 shed*) OR infectiousness OR infectivity OR contagiousness OR ((viral or virus) ADJ5 (emission* or emit*)) OR ((duration OR days OR time) ADJ5 shed*) OR (shed* ADJ5 (replica OR competent OR viable OR infecti*))).ti,ab,kf,hw. 3. 1 OR 2 4. exp Polymerase Chain Reaction/ OR exp clinical laboratory techniques/ 5. (polymerase chain reaction OR PCR OR RT-PCR OR RT-qPCR OR qPCR OR swab* OR nasal OR nasopharyngeal OR specimen* OR sample* OR test* OR assay* OR clinical technique* OR lab* OR cultures OR detect*).ti,ab,kf,hw. 6. 4 OR 5 7. (Symptom* OR fever* OR chill* OR cough* OR (difficult* ADJ2 breathing) OR (shortness ADJ2 breath*) OR dyspnea OR aches OR fatigue OR headache OR sore throat OR congestion OR runny nose OR taste OR ageusia OR smell OR anosmia OR nausea OR vomiting OR diarrhea).mp 8. 3 AND 6 9. Limit 8 to covid-19 10. limit 9 to ed="20240123-20240325" 11. 3 AND 7 12. Limit 11 to COVID-19 13. limit 12 to yr="2024 -Current" 14. 10 OR 13 15. Omicron.mp 16. 14 AND 15 |
| **Embase**  **(OVID)**  **1974-** | 1. Virus Shedding/ 2. (((viral OR virus) ADJ2 shed*) OR infectiousness OR infectivity OR contagiousness OR ((viral or virus) ADJ5 (emission* or emit*)) OR ((duration OR days OR time) ADJ5 shed*) OR (shed* ADJ5 (replica OR competent OR viable OR infecti*))).ti,ab,kf,hw. 3. 1 OR 2 4. exp Polymerase Chain Reaction/ OR exp laboratory technique/ 5. (polymerase chain reaction OR PCR OR RT-PCR OR RT-qPCR OR qPCR OR swab* OR nasal OR nasopharyngeal OR specimen* OR sample* OR test* OR assay* OR clinical technique* OR lab* OR cultures OR detect*).ti,ab,kf,hw. 6. 4 OR 5 7. (Symptom* OR fever* OR chill* OR cough* OR (difficult* ADJ2 breathing) OR (shortness ADJ2 breath*) OR dyspnea OR aches OR fatigue OR headache OR sore throat OR congestion OR runny nose OR taste OR ageusia OR smell OR anosmia OR nausea OR vomiting OR diarrhea).mp 8. 3 AND 6 9. Limit 8 to covid-19 10. limit 9 to dc="20240123-20240325" 11. 3 AND 7 12. Limit 11 to COVID-19 13. limit 12 to yr="2024 -Current" 14. 10 OR 13 15. Omicron.mp 16. 14 AND 15 |
| **Cochrane Library** | #1 [mh "Virus Shedding"]  #2 (((viral OR virus) NEAR/2 shed*) OR infectiousness OR infectivity OR contagiousness OR ((viral or virus) NEAR/5 (emission* or emit*)) OR ((duration OR days OR time) NEAR/5 shed*) OR (shed* NEAR/5 (replica OR competent OR viable OR infecti*))):ti,ab,kw  #3 #1 OR #2  #4 [mh ^"Polymerase Chain Reaction"] OR [mh ^"clinical laboratory techniques"]  #5 ("polymerase chain reaction" OR PCR OR "RT-PCR" OR "RT-qPCR" OR qPCR OR swab* OR nasal OR nasopharyngeal OR specimen* OR sample* OR test* OR assay* OR "clinical technique" OR "clinical techniques" OR lab* OR cultures OR detect*):ti,ab,kw  #6 #4 OR #5  #7 (Symptom* OR fever* OR chill* OR cough* OR (difficult* NEAR/2 breathing) OR (shortness NEAR/2 breath*) OR dyspnea OR aches OR fatigue OR headache OR "sore throat" OR congestion OR "runny nose" OR taste OR ageusia OR smell OR anosmia OR nausea OR vomiting OR diarrhea):ti,ab,kw  #8 #3 AND #6  #9 [mh "Covid-19"] OR (coronavir* OR "corona virus" OR covid19 OR covid OR nCoV OR "novel CoV" OR "CoV 2" OR CoV2 OR sarscov2 OR sars2 OR 2019nCoV OR NCOV19):ti,ab,kw  #10 #8 AND #9  #11 #3 AND #7 AND #9  #12 #10 OR #11  #13 omicron  #14 #12 AND #13  Limit to January 2024-current |
| **CINAHL**  **(EBSCOHost)** | S1 (MH "Virus Shedding")  S2 (((viral OR virus) N2 shed*) OR infectiousness OR infectivity OR contagiousness OR ((viral or virus) N5 (emission* or emit*)) OR ((duration OR days OR time) N5 shed*) OR (shed* N5 (replica OR competent OR viable OR infecti*)))  S3 S1 OR S2  S4 (MH "Polymerase Chain Reaction+") OR (MH "clinical laboratory techniques+")  S5 ("polymerase chain reaction" OR PCR OR "RT-PCR" OR "RT-qPCR" OR qPCR OR swab* OR nasal OR nasopharyngeal OR specimen* OR sample* OR test* OR assay* OR "clinical technique" OR "clinical techniques" OR lab* OR cultures OR detect*)  S6 S4 OR S5  S7 (Symptom* OR fever* OR chill* OR cough* OR (difficult* N2 breathing) OR (shortness N2 breath*) OR dyspnea OR aches OR fatigue OR headache OR "sore throat" OR congestion OR "runny nose" OR taste OR ageusia OR smell OR anosmia OR nausea OR vomiting OR diarrhea)  S8 S3 AND S6  S9 (MH "COVID-19+") OR (TI (coronavir* OR "corona virus*" OR covid19 OR covid OR nCoV OR "novel CoV" OR "CoV 2" OR CoV2 OR sarscov2 OR sars2 OR 2019nCoV OR NCOV19)) OR (AB (coronavir* OR "corona virus*" OR covid19 OR covid OR nCoV OR "novel CoV" OR "CoV 2" OR CoV2 OR sarscov2 OR sars2 OR 2019nCoV OR NCOV19))  S10 S8 AND S9  S11 S3 AND S7 AND S9  S12 S10 OR S11  S13 omicron  S14 S12 AND S13  **Limiters** - Published Date: 20240101-20240325; Exclude MEDLINE records |
| **Scopus** | (INDEXTERMS("Virus Shedding") OR TITLE-ABS-KEY(((viral OR virus) W/2 shed*) OR infectiousness OR infectivity OR contagiousness OR ((viral or virus) W/5 (emission* or emit*)) OR ((duration OR days OR time) W/5 shed*) OR (shed* W/5 (replica OR competent OR viable OR infecti*)))) AND (INDEXTERMS(("Polymerase Chain Reaction") OR ("laboratory techniques")) AND TITLE-ABS-KEY("polymerase chain reaction" OR PCR OR "RT-PCR" OR "RT-qPCR" OR qPCR OR swab* OR nasal OR nasopharyngeal OR specimen* OR sample* OR test* OR assay* OR "clinical technique" OR "clinical techniques" OR lab* OR cultures OR detect*)) AND (INDEXTERMS(COVID-19) OR TITLE-ABS-KEY(coronavir* OR "corona virus*" OR covid19 OR covid OR nCoV OR "novel CoV" OR "CoV 2" OR CoV2 OR sarscov2 OR sars2 OR 2019nCoV OR NCOV19)) AND NOT INDEX(medline)  OR  (INDEXTERMS("Virus Shedding") OR TITLE-ABS-KEY(((viral OR virus) W/2 shed*) OR infectiousness OR infectivity OR contagiousness OR ((viral or virus) W/5 (emission* or emit*)) OR ((duration OR days OR time) W/5 shed*) OR (shed* W/5 (replica OR competent OR viable OR infecti*))) AND TITLE-ABS-KEY(Symptom* OR fever* OR chill* OR cough* OR (difficult* W/2 breathing) OR (shortness W/2 breath*) OR dyspnea OR aches OR fatigue OR headache OR "sore throat" OR congestion OR "runny nose" OR taste OR ageusia OR smell OR anosmia OR nausea OR vomiting OR diarrhea)) AND (INDEXTERMS(COVID-19) OR TITLE-ABS-KEY(coronavir* OR "corona virus*" OR covid19 OR covid OR nCoV OR "novel CoV" OR "CoV 2" OR CoV2 OR sarscov2 OR sars2 OR 2019nCoV OR NCOV19)) AND NOT INDEX(medline)  AND  TITLE-ABS-KEY(omicron*)  AND   ( LIMIT-TO ( PUBYEAR , 2024 ) ) |

**Table s7.** Search Strategies for RQ2.b: Influenza Shedding & Symptom Association, Run July 26, 2024

| **Database** | **Strategy** |
| --- | --- |
| **Medline**  **(OVID)**  **1946-** | 1. exp Influenza, Human/ 2. (influenza* OR flu).ti,ab,kf,hw. 3. 1 OR 2 4. exp virus shedding/ 5. ((shed* OR replica* OR competent OR infectiousness OR infectivity OR contagiousness emission* or emit* OR viable OR viability) ADJ5 (duration OR period OR time OR timing OR cease OR cessation)) .ti,ab,kf,hw. 6. 4 OR 5 7. (Symptom* OR fever* OR chill* OR cough* OR (difficult* ADJ2 breathing) OR (shortness ADJ2 breath*) OR dyspnea OR aches OR fatigue OR headache OR sore throat OR congest* OR runny nose OR stuffy nose OR nausea OR vomiting OR diarrhea).mp 8. 3 AND 6 AND 7 |
| **Embase**  **(OVID)**  **1974-** | 1. exp Influenza/ 2. (influenza* OR flu).ti,ab,kf,hw. 3. 1 OR 2 4. exp virus shedding/ 5. ((shed* OR replica* OR competent OR infectiousness OR infectivity OR contagiousness emission* or emit* OR viable OR viability) ADJ5 (duration OR period OR time OR timing OR cease OR cessation)).ti,ab,kf,hw. 6. 4 OR 5 7. (Symptom* OR fever* OR chill* OR cough* OR (difficult* ADJ2 breathing) OR (shortness ADJ2 breath*) OR dyspnea OR aches OR fatigue OR headache OR sore throat OR congest* OR runny nose OR stuffy nose OR nausea OR vomiting OR diarrhea).mp 8. 3 AND 6 AND 7 9. Limit 8 to pubmed/medline 10. 8 NOT 9 |
| **Cochrane Library** | [mh "Influenza, Human"]  (influenza* OR flu):ti,ab,kw  #1 OR #2  [mh ^"virus shedding"]  ((shed* OR replica* OR competent OR infectiousness OR infectivity OR contagiousness OR emission* or emit* OR viable OR viability) NEAR/5 (duration OR period OR time OR timing OR cease OR cessation)):ti,ab,kw  #4 OR #5  #3 AND #6  (Symptom* OR fever* OR chill* OR cough* OR (difficult* NEAR/2 breathing) OR (shortness NEAR/2 breath*) OR dyspnea OR aches OR fatigue OR headache OR "sore throat" OR congest* OR "runny nose" OR "stuffy nose" OR nausea OR vomiting OR diarrhea):ti,ab,kw  #7 AND #8 |
| **CINAHL**  **(EBSCOHost)** | (MH "Influenza, Human")  (influenza* OR flu)  S1 OR S2  (MH "virus shedding")  ((shed* OR replica* OR competent OR infectiousness OR infectivity OR contagiousness OR emission* or emit* OR viable OR viability) N5 (duration OR period OR time OR timing OR cease OR cessation))  S4 OR S5  S3 AND S6  (Symptom* OR fever* OR chill* OR cough* OR (difficult* N2 breathing) OR (shortness N2 breath*) OR dyspnea OR aches OR fatigue OR headache OR "sore throat" OR congest* OR "runny nose" OR "stuffy nose" OR nausea OR vomiting OR diarrhea)  S7 AND S8  **Limiters** - Exclude MEDLINE records |
| **Scopus** | TITLE-ABS-KEY(influenza* OR flu) AND TITLE-ABS-KEY((shed* OR replica* OR competent OR infectiousness OR infectivity OR contagiousness OR emission* or emit* OR viable OR viability) W/5 (duration OR period OR time OR timing OR cease OR cessation)) AND TITLE-ABS-KEY(Symptom* OR fever* OR chill* OR cough* OR (difficult* N2 breathing) OR (shortness N2 breath*) OR dyspnea OR aches OR fatigue OR headache OR "sore throat" OR congest* OR "runny nose" OR "stuffy nose" OR nausea OR vomiting OR diarrhea) AND NOT INDEX(medline) |

**Table s8.** RQ3.a. (Omicron Serial Interval) Search Strategies, Run July 26, 2024

| **Database** | **Strategy** |
| --- | --- |
| **Medline**  **(OVID)**  **1946-** | 1. Serial Infection Interval/ 2. (Time factors/ AND (transmission OR ts.fs)) 3. (Serial interval* OR serial infection interval* OR generation time* OR generation interval* OR time series OR (transmission ADJ2 chain) OR (onset ADJ5 time) OR transmission dynamics OR (transmission ADJ2 characteristics) OR (time ADJ5 transmission)).ti,ab,kf. 4. 1 OR 2 OR 3 5. ((exp SARS-CoV-2/ OR exp Covid-19/ OR SARS* OR COVID* OR coronavirus*) AND omicron.mp.) 6. 4 AND 5 |
| **Embase**  **(OVID)**  **1974-** | 1. serial interval/ 2. ((Time factor/ OR Time/) AND transmission*.ti,ab,kf,hw.) 3. (Serial interval* OR serial infection interval* OR generation time* OR generation interval* OR time series OR (transmission ADJ2 chain) OR (onset ADJ5 time) OR transmission dynamics OR (transmission ADJ2 characteristics) OR (time ADJ5 transmission)).ti,ab,kf. 4. 1 OR 2 OR 3 5. "SARS-CoV-2 Omicron (lineage BQ.1.1)"/ or SARS-CoV-2 Omicron/ or omicron.mp. 6. 4 AND 5 |
| **Cochrane Library** | 1. [mh "serial infection interval"] OR (serial NEAR/2 interval*) OR (generation NEAR/2 time*) OR (generation NEAR/2 interval*) OR (time NEAR/2 series) OR (transmission NEAR/2 chain) OR (onset NEAR/2 time) OR "transmission dynamics" OR (transmission NEAR/2 characteristics) OR (time NEAR/5 transmission) 2. omicron* 3. #1 AND #2 |
| **CINAHL**  **(EBSCOHost)** | (MH "Serial Infection Interval") OR (serial N2 interval*) OR "generation time*" OR "generation interval*" OR "time series" OR (transmission N2 chain) OR (onset N2 time) OR "transmission dynamics" OR (transmission N2 characteristics) OR (time N5 transmission)  omicron*  S1 AND S2  **Limiters** - Published Date: 20200101-20231231 |
| **Scopus** | TITLE-ABS-KEY((serial W/2 interval*) OR "generation time*" OR "generation interval*" OR "time series" OR (transmission W/2 chain) OR (onset W/2 time) OR "transmission dynamics" OR (transmission W/2 characteristics) OR (time W/5 transmission)) AND TITLE-ABS-KEY(omicron*) AND TITLE-ABS-KEY(SARS* OR COVID* OR Coronavirus*) AND NOT INDEX(medline) |

**Table s9.** Search Strategies for RQ3.b: Influenza Serial Interval, run on July 26, 2024

| **Database** | **Strategy** |
| --- | --- |
| **Medline**  **(OVID)**  **1946-** | 1. Serial Infection Interval/ 2. (Time factors/ AND (transmission OR ts.fs)) 3. (Serial interval* OR serial infection interval* OR generation time* OR generation interval* OR time series OR (transmission ADJ2 chain) OR (onset ADJ5 time) OR transmission dynamics OR (transmission ADJ2 characteristics) OR (time ADJ5 transmission)).ti,ab,kf. 4. 1 OR 2 OR 3 5. Exp Influenza, Human/tm OR *Influenza, Human/ OR influenz*.ti,ab,kf. 6. 4 AND 5 |
| **Embase**  **(OVID)**  **1974-** | 1. serial interval/ 2. ((Time factor/ OR Time/) AND transmission*.ti,ab,kf,hw.) 3. (Serial interval* OR serial infection interval* OR generation time* OR generation interval* OR time series OR (transmission ADJ2 chain) OR (onset ADJ5 time) OR transmission dynamics OR (transmission ADJ2 characteristics) OR (time ADJ5 transmission)).ti,ab,kf. 4. 1 OR 2 OR 3 5. Exp Influenza/ OR exp influenza virus/ OR influenz*.mp 6. 4 AND 5 7. limit 6 to "pubmed/medline" 8. 6 NOT 7 |
| **Cochrane Library** | 1. [mh "serial infection interval"] OR (serial NEAR/2 interval*) OR (generation NEAR/2 time*) OR (generation NEAR/2 interval*) OR (time NEAR/2 series) OR (transmission NEAR/2 chain) OR (onset NEAR/2 time) OR "transmission dynamics" OR (transmission NEAR/2 characteristics) OR (time NEAR/5 transmission) 2. [mh ^"Influenza, Human"] OR influenz*:ti,ab,kw 3. #1 AND #2 |
| **CINAHL**  **(EBSCOHost)** | 1. (MH "Serial Infection Interval") OR (serial N2 interval*) OR "generation time*" OR "generation interval*" OR "time series" OR (transmission N2 chain) OR (onset N2 time) OR "transmission dynamics" OR (transmission N2 characteristics) OR (time N5 transmission) 2. (MH "Influenza, Human+") OR (TI influenz*) OR (AB influenz*) 3. S1 AND S2 |
| **Scopus** | TITLE-ABS-KEY((serial W/2 interval*) OR "generation time*" OR "generation interval*" OR "time series" OR (transmission W/2 chain) OR (onset W/2 time) OR "transmission dynamics" OR (transmission W/2 characteristics) OR (time W/5 transmission)) AND TITLE-ABS-KEY(influenz*) AND NOT INDEX(medline) |

## **Part IV.B PRISMA Diagrams**

**Figure s7.** Prisma Diagram for KQ1.a Omicron Shedding

**Identification**

References from other sources **(n = 0)**

Citation searching (n = 0)

Grey literature (n = 0)

Studies screened **(n = 4,924)**

Studies sought for retrieval **(n = 722)**

Studies assessed for eligibility **(n = 722)**

References removed **(n = 271)**

Duplicates identified by Covidence (n = 268)

Duplicates identified manually (n = 3)

Studies excluded **(n = 4,202)**

Studies not retrieved **(n = 0)**

**Included**

Studies included in review **(n = 8)**

**Screening**

Studies from databases/registers **(n = 5,195)**

MEDLINE (n = 3327)

Embase (n = 1624)

Scopus (n = 68)

Cochrane Library (n = 118)

CINAHL (n = 58)

Studies excluded **(n = 714)**

Duplicate (n = 4)

Not in English (n = 11)

No primary data (n = 31)

No population of interest (n = 28)

No intervention of interest (n = 368)

No outcome of interest (n = 221)

Not able to verify complete methods (n = 51)

**Figure s8.** Prisma Diagram for KQ1.b Influenza Shedding

**Identification**

References from other sources **(n = 0)**

Citation searching (n =0 )

Grey literature (n =0 )

Studies screened **(n = 1,432)**

Studies sought for retrieval **(n = 162)**

Studies assessed for eligibility **(n = 162)**

References removed **(n =31)**

Duplicates identified manually (n = 25)

Duplicates identified by Covidence (n = 6)

Studies excluded **(n = 1,270)**

Studies not retrieved **(n = 0)**

**Included**

Studies included in review **(n = 6)**

**Screening**

Studies from databases/registers **(n = 1,463)**

MEDLINE (n = 909)

Embase (n = 452)

Scopus (n = 53)

Cochrane Library (n = 45)

CINAHL (n = 4)

Studies excluded **(n = 156)**

Not in English (n = 6)

No primary data (n = 7)

No population of interest (n = 33)

No intervention of interest (n = 22)

No outcomes of interest (n = 81)

Not able to verify complete methods (n = 7)

**Figure s9.** Prisma Diagram for KQ2.a Omicron Shedding & Symptom Association

**Identification**

References from other sources **(n = 0)**

Citation searching (n = 0)

Grey literature (n = 0)

Studies screened **(n = 793)**

Studies sought for retrieval **(n = 174)**

Studies assessed for eligibility **(n = 174)**

References removed **(n = 7)**

Duplicates identified manually (n =3)

Duplicates identified by Covidence (n =4)

Studies excluded **(n = 619)**

Studies not retrieved **(n = 0)**

**Included**

Studies included in review **(n = 1)**

**Screening**

Studies from databases/registers **(n = 800)**

MEDLINE (n = 474)

Embase (n = 316)

Scopus (n = 5)

Cochrane Library (n = 4)

CINAHL (n = 1)

Studies excluded **(n = 173)**

Not in English (n = 1)

No primary data (n = 4)

No outcome of interest (n = 159)

No population of interest (n = 8)

Not able to verify complete methods (n = 1)

**Figure s10.** Prisma Diagram for KQ2.b Influenza Shedding & Symptom Association

**Identification**

References from other sources **(n = 9)**

Citation searching (n = 9)

Grey literature (n = 0)

Studies screened **(n = 977)**

Studies sought for retrieval **(n = 171)**

Studies assessed for eligibility **(n = 171)**

References removed **(n = 3)**

Duplicates identified manually (n = 2)

Duplicates identified by Covidence (n = 1)

Studies excluded **(n = 806)**

Studies not retrieved **(n = 0)**

**Included**

Studies included in review **(n = 3)**

**Screening**

Studies from databases/registers **(n = 971)**

MEDLINE (n = 274)

Embase (n = 646)

Scopus (n = 16)

Cochrane Library (n = 35)

CINAHL (n = 0)

Studies excluded **(n = 168)**

No primary data (n = 3)

Not in English (n = 4)

No population of interest (n = 12)

No intervention of interest (n = 2)

No outcomes of interest (n = 126)

Not able to verify complete methods (n = 21)

**Figure s11.** Prisma Diagram for KQ3.a Omicron Serial Interval

**Identification**

References from other sources **(n = 0)**

Citation searching (n = 0)

Grey literature (n = 0)

Studies screened **(n = 269)**

Studies sought for retrieval **(n = 72)**

Studies assessed for eligibility **(n = 72)**

References removed **(n = 3)**

Duplicates identified manually (n = 1)

Duplicates identified by Covidence (n = 2)

Studies excluded **(n = 197)**

Studies not retrieved **(n = 0)**

**Included**

Studies included in review **(n = 14)**

**Screening**

Studies from databases/registers **(n = 272)**

MEDLINE (n = 125)

Embase (n = 78)

Scopus (n = 53)

Cochrane Library (n = 10)

CINAHL (n = 6)

Studies excluded **(n = 58)**

Duplicate (n = 1)

Not primary data (n = 1)

Not written in English (n = 5)

No outcomes of interest (n = 50)

Not able to verify complete methods (n = 1)

**Figure s12.** Prisma Diagram for KQ3.b Influenza Serial Interval

**Identification**

References from other sources **(n = 4)**

Citation searching (n = 4)

Grey literature (n = 0)

Studies screened **(n = 3,248)**

Studies sought for retrieval **(n = 123)**

Studies assessed for eligibility **(n = 123)**

References removed **(n = 37)**

Duplicates identified manually (n = 2)

Duplicates identified by Covidence (n = 35)

Studies excluded **(n = 3,125)**

Studies not retrieved **(n = 0)**

**Included**

Studies included in review **(n = 14)**

**Screening**

Studies from databases/registers **(n = 3,281)**

MEDLINE (n = 2052)

Embase (n = 775)

Scopus (n = 337)

Cochrane Library (n = 57)

CINAHL (n = 60)

Studies excluded **(n = 109)**

No primary data (n = 8)

Not written in English (n = 5)

No population of interest (n = 20)

No outcomes of interest (n = 69)

Not able to verify complete methods (n = 7)
